# Loss of LncRNA DUXAP8 synergistically enhanced sorafenib induced ferroptosis in hepatocellular carcinoma via SLC7A11 de‐palmitoylation

**DOI:** 10.1002/ctm2.1300

**Published:** 2023-06-19

**Authors:** Zhehao Shi, Zhiming Li, Bin Jin, Wen Ye, Luhui Wang, Sina Zhang, Jiuyi Zheng, Zixia Lin, Bo Chen, Fangting Liu, Baofu Zhang, Xiwei Ding, Zhen Yang, Yunfeng Shan, Zhengping Yu, Yi Wang, Jicai Chen, Qiang Chen, Lewis R. Roberts, Gang Chen

**Affiliations:** ^1^ Department of Dermatology The First Affiliated Hospital of Wenzhou Medical University Wenzhou China; ^2^ Key Laboratory of Diagnosis and Treatment of Severe Hepato‐Pancreatic Diseases of Zhejiang Province The First Affiliated Hospital of Wenzhou Medical University Wenzhou Zhejiang China; ^3^ Department of Organ Transplantation Qilu Hospital Cheeloo College of Medicine Shandong University Shandong China; ^4^ Department of Hepatobiliary Surgery The Second Hospital of Shandong University Shandong China; ^5^ Department of Breast Surgery The Second Affiliated Hospital and Yuying Children's Hospital of Wenzhou Medical University Wenzhou China; ^6^ Department of Gastroenterology The Affiliated Drum Tower Hospital of Nanjing University Medical School Nanjing China; ^7^ Department of Infectious Diseases Shandong Provincial Hospital affiliated to Shandong University Jinan China; ^8^ Department of Hepatobiliary Surgery The First Affiliated Hospital of Wenzhou Medical University Wenzhou China; ^9^ Department of Epidemiology and Biostatistics School of Public Health and Management Wenzhou Medical University Wenzhou China; ^10^ Department of Hernia and Abdominal Wall Surgery The First Affiliated Hospital of Wenzhou Medical University Wenzhou China; ^11^ Cancer Centre Faculty of Health Sciences University of Macau Macau, Arkansas China; ^12^ MOE Frontier Science Centre for Precision Oncology University of Macau, Taipa Macau, Arkansas China; ^13^ Division of Gastroenterology and Hepatology Mayo Clinic College of Medicine and Science Rochester Minnesota USA

**Keywords:** DUXAP8, ferroptosis, hepatocellular carcinoma, sorafenib resistance

## Abstract

**Background:**

Ferroptosis is an important iron‐dependent form of cell death in hepatocellular carcinoma (HCC). Sorafenib, a potent ferroptosis inducer, is used to treat advanced HCC but its efficacy is limited by the development of drug resistance.

**Methods:**

The effects of DUXAP8 expression on HCC progression were evaluated by TCGA database, Kaplan‐Meier analysis, and in situ hybridization analysis. Sorafenib resistant HCC cell lines were modeled in vitro to study the regulation of DUXAP8 on ferroptosis in HCC induced by sorafenib. We used RNA pull‐down, immunofluorescence assays, acyl‐biotinyl exchange assay and mass spectrometry analysis to assess the molecular mechanism of ferroptosis regulation by DUXAP8. Syngeneic subcutaneous and orthotopic CDX models were used to assess whether DUXAP8 inhibition improves HCC in vivo.

**Results:**

LncRNA DUXAP8, which is highly expressed in liver cancer and associated with poor prognosis, contributes to sorafenib resistance through suppression of ferroptosis. In vitro tests revealed that DUXAP8 reduced the sensitivity of HCC to sorafenib‐induced ferroptosis by acting on SLC7A11, a subunit of the amino acid antiporter system xc‐. DUXAP8 facilitates SLC7A11 palmitoylation and impedes its lysosomal degradation, thereby enhancing SLC7A11 action and suppressing ferroptosis. RNA pull‐down and immunofluorescence assays confirmed that DUXAP8 decreased membrane translocation and promoted sorting of de‐palmitoylated SLC7A11 to lysosomes by binding of DUXAP8 to SLC7A11. In addition, mass spectrometric analysis found that the Cys414 residue of SLC7A11 might be the predominant mutant site responsible for molecular masking of SLC7A11 lysosomal sorting. Further, the antitumor effect of DUXAP8 knockdown was verified in orthotopic and subcutaneous CDX models.

**Conclusions:**

Our findings suggest that a novel translational strategy combining sorafenib with DUXAP8 silencing to overcome drug resistance may improve treatment efficacy in patients with advanced HCC.

## Graphical abstract

- DUXAP8 mediated activation of SLC7A11 restrained the sensitivity of HCC to sorafenib induced ferroptosis.
- DUXAP8 facilitated the previously unknown palmitoylation of SLC7A11 and prevented SLC7A11 lysosomal degradation.
- Loss of LncRNA DUXAP8 synergistically enhanced sorafenib induced ferroptosis in HCC.

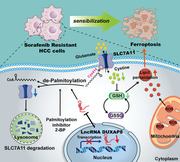

## 1 BACKGROUND

Primary liver cancer ranked fifth globally in terms of cancer incidence and was the third leading cause of cancer‐related death in 2020. Hepatocellular carcinoma (HCC), which accounts for 75%−85% of primary liver cancer, is diagnosed in more than 700,000 persons each year.^1^, ^2^ In recent years, significant progress has been made in the treatment of HCC, including improved techniques for surgical resection, tumor ablation, loco‐regional therapy, targeted drug therapy, and immunotherapy.^2^, ^3^, ^4^, ^5^ Sorafenib, a multi‐targeted tyrosine kinase inhibitor that plays an important role in promoting apoptosis, inducing ferroptosis, reducing angiogenesis and inhibiting tumor cell proliferation, was approved by the FDA in 2007 as a first‐line drug for the treatment of HCC, demonstrating clear clinical benefit in advanced HCC patients.^5^, ^6^ However, the efficacy of sorafenib is limited by drug resistance, leading to a dissatisfactory 5‐year overall survival rate of advanced HCC patients.^7^, ^8^ Novel checkpoint inhibitor‐based regimens extend patient survival but produce durable responses in only 20−30% of patients. Receptor tyrosine kinase inhibitors, therefore, remain an essential component of the therapeutic armamentarium in HCC.^2^, ^9^, ^10^ Therefore, there is an urgent need to conceptualize new strategies for enhancing the effectiveness of systemic HCC treatments.

System x_c_^−^ is an amino acid antiporter that simultaneously transports cystine into cells in exchange for glutamate to synthesize the antioxidant glutathione (GSH),^11^which plays a vital role in ferroptosis.^12^, ^13^ Solute carrier family 7 membrane 11 (SLC7A11) is the light chain subunit of system x_c_^−^, and its expression level is positively correlated with the activity of the antiporter.^14^ Ferroptosis inducers, including eradicator of ST (erastin) and sorafenib, can reduce antioxidant capacity and decrease cysteine amino acid metabolites, leading to ferroptosis of HCC cells.^15^, ^16^This suggests the possible involvement of SLC7A11 in sorafenib resistance via ferroptosis. Nevertheless, the mechanism underlying the regulation of SLC7A11 expression and its translocation to the HCC cell membrane remains unclear.

Long noncoding RNAs (lncRNAs) have been linked to the pathogenesis of various human tumors^17^, ^18^ and shown to interact with proteins, regulating cancer signaling pathways through post‐translational modifications.^19^ Double homeobox A pseudogene 8 (DUXAP8), a pseudogene‐derived lncRNA, may play a promoting role in pancreatic cancer,^20^ non‐small‐cell lung cancer,^21^ and HCC through actions on the Fork head Box M1 (FoxM1) protein.^22^ However, the role of DUXAP8 in sorafenib's therapeutic effect and drug resistance in HCC treatment remains to be clarified.

In this study, we confirmed that the expression of DUXAP8 was closely correlated to the malignant phenotype and prognosis of HCC. Through effects on SLC7A11 protein levels, DUXAP8 deficiency enhanced ferroptosis and sensitized HCC cells to sorafenib. Our findings reveal a new mechanistic axis involving lncRNAs and SLC7A11 in ferroptosis regulation.

## 2 METHODS

### 2.1 Antibodies and reagents

The primary antibodies for SLC7A11 (12691 (Cell Signaling Technology), ab175186 (Abcam) and 26864‐1‐AP (Proteintech), Flag tag (14793; Cell Signaling Technology), ACSL4 (ab155282; Abcam), Streptavidin (3419; Cell Signaling Technology), GPX4(ab125066; Abcam), LAMP2 (66301‐1‐Ig; Proteintech), Anti‐4Hydroxynonenal (ab48506; Abcam), GAPDH (ab8245; Abcam), andβ‐actin (ab8226; Abcam) were commercially available. The following secondary antibodies were used in immunofluorescence assays: AF488‐anti‐Mouse (Invitrogen), AF594‐anti‐rabbit (Invitrogen), AF594‐anti‐mouse (Invitrogen), AF568‐anti‐rabbit (Abcam), AF647‐anti‐rabbit (Abcam), and AF647‐anti‐mouse (Abcam). Small‐molecular compounds such as Sorafenib (HY‐10201), Erastin (HY‐15763), Bafilomycin (HY‐100558), MG132 (HY‐13259), Cycloheximide (HY‐12320), Chlorquinaldol (HY‐17589A), Z‐VAD‐FMK(HY‐16658B), Necrosulfonamide (HY‐100573) and Ferrostatin‐1 (HY‐100579) were all from MCE. 2‐BP (21604) was from Sigma–Aldrich.

### 2.2 Patients and clinical tissue samples

From the Department of Hepatobiliary Surgery at the First Affiliated Hospital of Wenzhou University, two cohorts of hepatocellular carcinoma tissues samples were obtained. All samples analyzed in this study were confirmed as hepatocellular carcinoma by pathology. Cohort 1 contains 20 cases of fresh frozen specimens, including HCC tissues and paired benign tumor‐adjacent tissues (>2 cm away from the resection margin), collected during the period between November 2018 and January 2019. Cohort 2 contains paraffin‐embedded HCC and adjacent tissue samples with detailed clinical information from 109 patients diagnosed between May 2016 and October 2019. Pathologists with expertise graded postoperative tumor‐lymph node‐metastasis (TNM) staging and Edmondson‐Steiner grading.

### 2.3 Acyl‐biotinyl exchange (ABE) assay and mass spectrometry analysis

The lysis buffer (50 mM Tris‐HCL, pH7.5, 150 mM NaCl, 1 mM EDTA, 1%(v/v) Triton X‐100) supplied with complete protease inhibitor was prepared for cell lysis. The cell lysate was harvested subjected to immunoprecipitation with Anti‐Flag Sepharose Beads (Millipore, A2220) and Anti‐IgG Sepharose Bead (CST, 3420) for 2 h at 4 °C. After centrifuging, the protein pellets were separated into two halves and rolling‐over in the lysis buffer containing N‐ethylmaleimide at RT for 30 min. Then, one portion was overturned with hydroxylamine buffer at RT for 1 h to cleave the thioester bonds of the palmitoylation. Another with standard lysis buffer was prepared as a control. Next, HA‐biotin labels the newly exposed cysteinyl thiols, and Streptavidin‐HRP affinity purification of biotinylated proteins. Finally, the pull‐down products were subjected to SDS–PAGE, identification of palmitoylated protein by Western blot and palmitoylation site by mass spectrometry analysis (AIMS Mass, CHN).^23^

### 2.4 Animal experiments

Mice were housed at the Laboratory Animal Center of First Affiliated Hospital of Wenzhou University. To establish an orthotopic cell line‐derived xenograft (CDX) model, the LM3 cells were transfected with lentivirus carrying luciferase and sh‐DUXAP8 or sh‐NC plasmids, screened with puromycin to obtain stable luciferase clones and amplified in vitro. Under pentobarbital sodium anesthesia, each nude mouse was injected intrahepatically with 20 μL of a mixture of PBS and Matrigel (1:5) containing 1 × 10^6^ cells. After the operation, the fluorescence intensity was evaluated weekly by IVIS‐SPECTRUM in vivo imaging system (PerkinElmer, US). Mice were executed after 21 days of treatment. 1×10^6^ LM3 cells transfected with sh‐DUXAP8 or sh‐NC were subcutaneously injected into two groups of 6 male nude mice, respectively. From the 6th day, the tumor growth was examined every 3 days, and the tumor volume was recorded and calculated following V = 1/2 (width2× length). 21 days after implantation, all mice were sacrificed under anaesthesia, and mice weights, tumor weights and volumes were determined. 1×10^6^ SR cells transfected with sh‐DUXAP8 or sh‐NC were subcutaneously injected into four groups of 7 male nude mice, respectively. From the 10th day, the sorafenib (30 mg/kg/each) or oral vehicle was administered by gavage every day depending on the mice group, and the tumor growth was examined every 2 days. Sorafenib was mixed in an oral vehicle containing castor oil, anhydrous ethanol and water in a ratio of 1:1:6. 24 days after implantation, all mice were sacrificed under anaesthesia, and mice weights, tumor weights and volumes were determined.

### 2.5 Statistical analysis

R program (version 4.0.2) and GraphPad Prism 8.0 software (GraphPad Software, Inc.) were used to conduct the statistical analyses. Data were presented as frequency (%) or mean ± standard deviation (SD). Comparisons of continuous variables between two groups were performed by Student's t‐test, whereas the chi‐square test or Fisher's exact test was used to compare categorical variables. One‐way analysis of variance (ANOVA) model was used to compare continuous outcomes across several experimental groups, and the Bonferroni technique was used for post hoc analysis. For survival analysis, Kaplan‐Meier survival curve techniques were utilized, and a log‐rank test was conducted to compare survival time between groups. Pearson correlation was performed to assess the correlation between expressions of two genes. Statistical significance was defined as a p‐value less than 0.05. Additional materials and methods can be found in Additional file: Supplementary Methods.

## 3 RESULTS

### 3.1 DUXAP8 expression correlates with malignant behavior and outcomes of HCC

**FIGURE 1:**
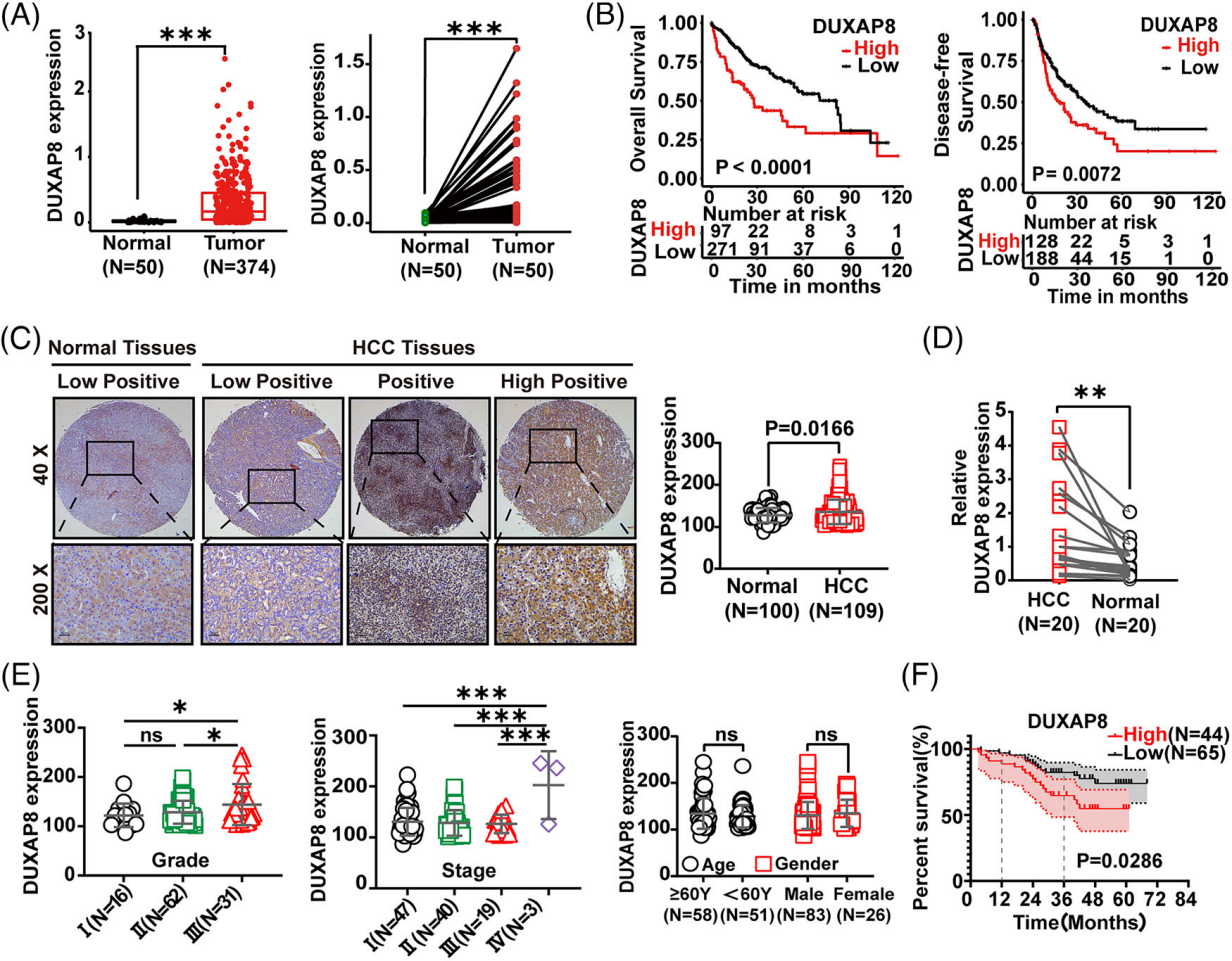
LncRNA DUXAP8 upregulation and its correlation with poor prognosis in HCC. (A) Expression of DUXAP8 was compared using the total sample data (374 HCC and 50 normal tissues) (Left) and the matched sample data (50 pairs of HCC and adjacent normal liver tissues from the same patient) (Right) extracted from the TCGA database. (B) Correlation of the DUXAP8 expression level with overall survival (OS) (Left, n = 368) and disease‐free survival (DFS) (Right, n = 316) in HCC patients from the TCGA database. (C) Expression of DUXAP8 was compared using the data of 109 HCC samples and 100 normal liver tissues obtained in our hospital. Left: representative in‐situ hybridization (ISH) images of DUXAP8. Magnification: ×40 (top panel) and ×200 (bottom panel). DUXAP8 expression was classified into three levels: low positive, positive, and high positive. Right: DUXAP8 staining scores in HCC and adjacent non‐tumor tissues. (D) Comparison of DUXAP8 expression in 20 pairs of HCC and adjacent normal liver tissues by qRT‐PCR. (E) Analysis of DUXAP8 expression in HCC tissues was stratified by pathological differentiation grade (Left), TNM stage (Middle), age and gender (Right). (F) Kaplan‐Meier curves of overall survival of 109 HCC patients with low (n = 65) and high (n = 44) DUXAP8 expression levels, the median value was used to subclassify HCCs into high‐ and low‐ expression groups (1‐year: 80.6% vs.89.2%; 3‐year: 64.7% vs.82.2%; p = 0.0286). *p < 0.05, ** *p* < 0.01, *** *p* < 0.001. NS: not significant.

Using data from the TCGA project, 374 HCC and 50 adjacent tissue samples, including 50 pairs of matching cancer and adjacent benign tissues, were analyzed for genomic expression and clinicopathologic characteristics (Figure 1A). DUXAP8 expression was significantly higher in HCC tissue than in adjacent benign tissue. Applying Kaplan–Meier (K‐M) survival analysis, higher DUXAP8 expression was associated with lower rates of overall survival (OS) and disease‐free survival (DFS) in HCC patients (Figure 1B). In addition, univariate and multivariate Cox regression analyses revealed that DUXAP8 expression level and cancer status were significantly associated with prognosis of HCC patients (Figure S1A,B).

109 HCC tissues and 100 adjacent benign tissues were used to assemble tissue microarrays and probed by in‐situ hybridization (ISH) assays for single‐molecule visualization of lncRNA DUXAP8. DUXAP8 expression was remarkably higher in HCC tissue than normal tissue (Figure 1C). The difference in DUXAP8 expression level between tumor and non‐tumor tissues was also confirmed by qRT‐PCR assay using 20 pairs of matching HCC and adjacent non‐tumor tissue samples (Figure 1D). In particular, the expression level of DUXAP8 was positively correlated with both the pathological grade and TNM stage of HCC, but there was no statistical association with gender and age (Figure 1E). Survival analysis confirmed that patients with higher DUXAP8 expression levels had a worse prognosis (Figure 1F).

In another set of experiments, qRT‐PCR confirmed that DUXAP8 expression was remarkably higher in the LM3 and HepaRG cells but lower in Huh7, PLC and Li‐7 cells (Figure S2A). Both knockdown and overexpression of DUXAP8 were successfully transfected (Figure S2B,C). DUXAP8 depletion prominently suppressed the growth of LM3 and HepaRG cells (Figure S2D), while DUXAP8 overexpression promoted the growth of Huh7 and Li‐7 cells (Figure S2E). Consistently, colony formation assays confirmed that DUXAP8 affects the proliferation of HCC cells (Figure S2F,G). Transwell assays showed that migration and invasion were impaired by DUXAP8 knockdown in the sh‐DUXAP8 group (Figures S2HandS2I), and enhanced by DUXAP8 overexpression (Figures S2JandS2K). DUXAP8 enhances the malignant phenotype of HCC cells.

### 3.2 Correlation between the expression of DUXAP8 and SLC7A11

**FIGURE 2:**
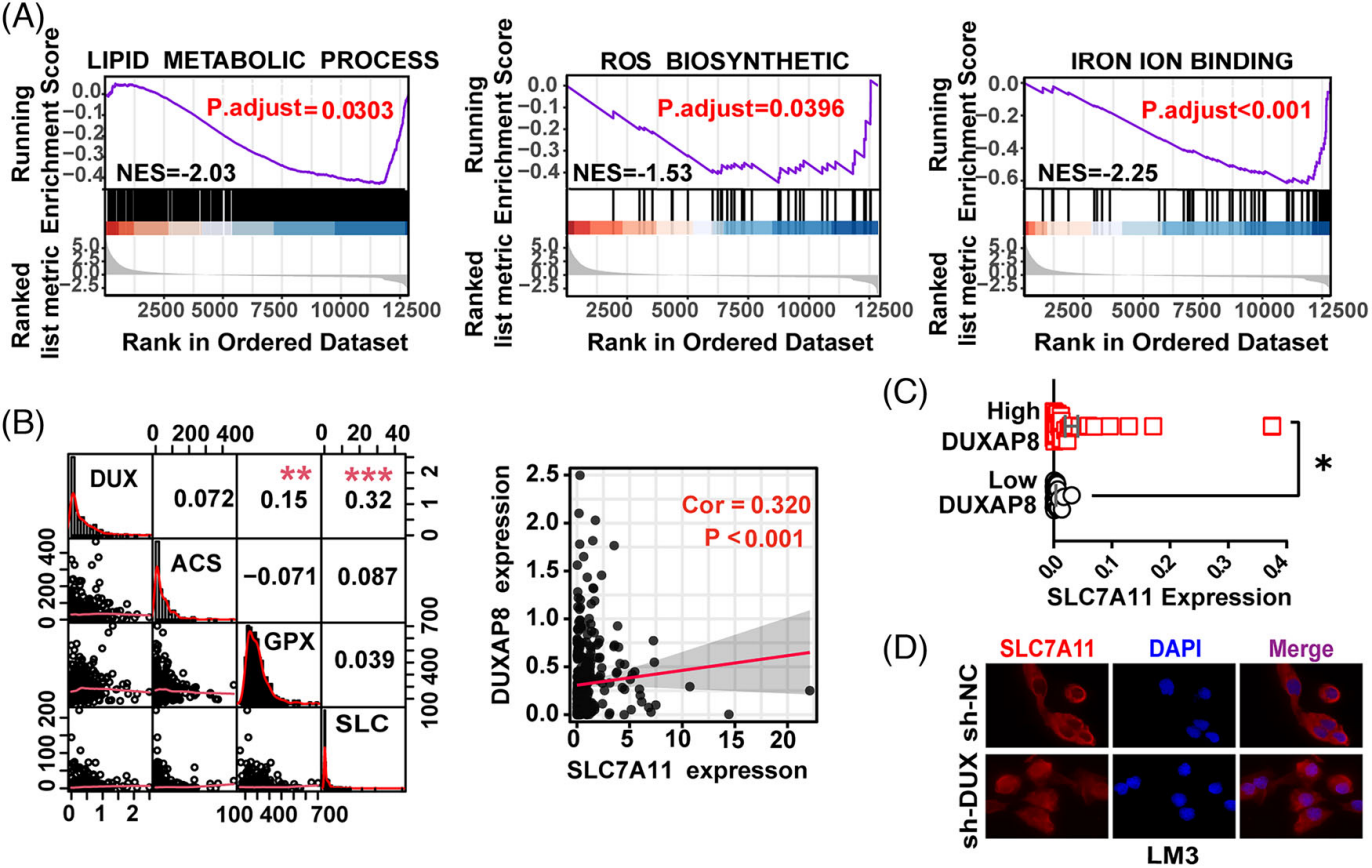
Construction of lncRNA DUXAP8 / SLC7A11 signaling pathway. (A) GSEA of the KEGG pathway analysis showing enrichment of DUXAP8 in the lipid metabolism (left), ROS biosynthesis (Middle) and iron ion binding (right). (B) Pearson correlation analysis between the expression of LncRNA DUXAP8 and key ferroptosis regulators ACSL4, GPX4, and SLC7A11 from TCGA (left). The expression of LncRNA DXUAP8 was positively correlated with SLC7A11 mRNA expression (right). (C) Comparison of SLC7A11 expression between low‐ (n = 65) and high‐DUXAP8 (n = 44) patients by qRT‐PCR. (D) Cellular localization of SLC7A11(Red) visualized by immunofluorescence in LM3 cells. Magnification: ×400. * *p* < 0.05. The results represent the mean value from three independent experiments. sh‐DUX: sh‐DUXAP8; DUX: DUXAP8; ACS: ACSL4; GPX: GPX4; SLC: SLC7A11.

The pathway network of DXUAP8 in HCC was constructed using data from the MSigDB database. Three pathways, including iron ion binding, lipid metabolism, and ROS biosynthesis, were downregulated in HCC (Figure 2A), implying a possible role of DUXAP8 in cell ferroptosis. Hence, an assessment of ferroptosis‐related mRNAs associated with DUXAP8 in HCC was conducted, and DUXAP8 expression had a closer positive relationship with SLC7A11 (Figures 2B and S3A). Among 109 HCC patients, SLC7A11 expression was remarkably lower in HCC tissue of the low‐DUXAP8 group compared to that of the high‐DUXAP8 group (Figure 2C). Next is investigating the effect of DUXAP8 on the subcellular localization of SLC7A11, which determines the main physiological functions of SLC7A11.^24^, ^25^ Immunofluorescence staining and Western blot (WB) assays showed that silencing DUXAP8 decreased the expression of SLC7A11 on the cell membrane (Figures 2D and S3B). WB assays also showed that DUXAP8 positively regulated SLC7A11 protein expression at the cellular level (Figure S3C–F). The results above suggested that the involvement of DUXAP8 in ferroptosis might be mediated by SLC7A11.

### 3.3 Role of DUXAP8 in erastin‐ and sorafenib‐induced ferroptosis

**FIGURE 3:**
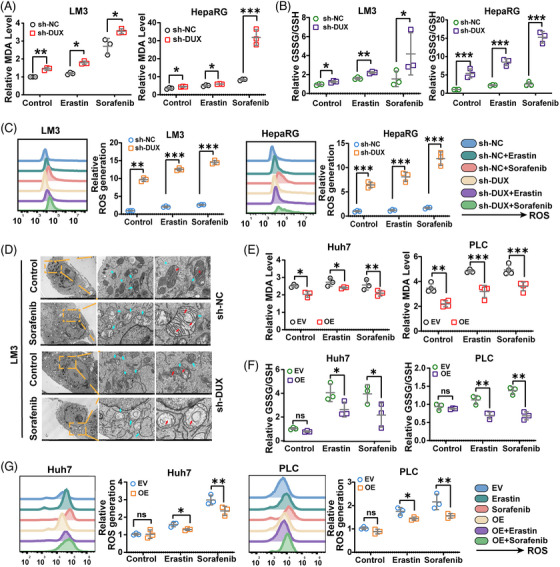
DUXAP8 regulates erastin and sorafenib induced ferroptosis in HCC cells. (A) DUXAP8 knockdown promoted the erastin and sorafenib induced lipid formation (measured by MDA assay) in LM3 (left) and HepaRG (right) cells treated with 10 μM erastin or sorafenib for 24 h. (B) DUXAP8 knockdown increased the GSSG/GSH ratios in erastin or sorafenib treated LM3 (left) and HepaRG (right) cells. (C) DUXAP8 knockdown increased the ROS level (assessed by DCFH‐DA staining) of LM3 cells (two on the left) and HepaRG cells (two on the right) treated with erastin (10 μM) or sorafenib (10 μM) for 24 h. (D) Representative TEM images showing sorafenib‐induced ferroptosis in LM3 cells with and without DUXAP8 knockdown. Scale bar: left, 5 μm; middle, 1 μm; right, 500 nm. (E) DUXAP8 overexpression suppressed the erastin and sorafenib induced lipid formation (measured by MDA assay) in Huh7 (left) and PLC (right) cells treated with 5 μM erastin or 2.5 μM sorafenib for 24 h. (F) DUXAP8 overexpression influenced the intracellular GSSG/GSH ratios in erastin or sorafenib treated Huh7 (left) and PLC (right) cells. (G) DUXAP8 overexpression inhibited the ROS level (assessed by DCFH‐DA staining) of Huh7 (two on the left) and PLC (two on the right) cells treated with erastin (5 μM) or sorafenib (2.5 μM) for 24 h. * *p* < 0.05, ** *p* < 0.01, *** *p* < 0.001. Results represent the average from three independent experiments. sh‐DUX, sh‐DUXAP8; EV, Empty vector; OE, pcDNA DUXAP8; sh‐SLC, sh‐SLC7A11; OE‐SLC, pcDNA SLC7A11.

Erastin and sorafenib induced cell ferroptosis in a dose‐dependent manner (Figure S4A–D). Suppression of DUXAP8 expression significantly increased cell death induced by erastin and sorafenib in the LM3 and HepaRG cells (Figure S4E,F). Next, the involvement of DUXAP8 in the accumulation of lipid peroxidation products, an indispensable signaling event during ferroptosis,^26^, ^27^ was clarified. DUXAP8 downregulation notably increased malondialdehyde (MDA) production, the ratio between oxidized glutathione and reduced glutathione (GSSG/GSH ratio), and the ROS level all induced by sorafenib and erastin in HCC cells (Figure 3A–C). Transmission electron microscopy (TEM) showed that mitochondrial cristae decreased or completely disappeared, and the outer membranes of mitochondria ruptured in LM3 cells treated with sorafenib; notably, the mitochondrial changes in ferroptosis were substantially enhanced by sh‐DUXAP8 transfection (Figure 3D).

The role of DUXAP8 in ferroptosis was also confirmed by overexpressing DUXAP8 in Huh7 and PLC cells. Erastin and sorafenib induced ferroptosis in Huh7 and PLC cells in a dose‐dependent manner (Figure S4G–J), and cell death could be mitigated by overexpression of DUXAP8 (Figure S4K,L). The levels of critical events in ferroptosis showed corresponding changes. As expected, the increases in MDA (Figure 3E), GSSG/GSH (Figure 3F), and ROS accumulation (Figure 3G) induced by erastin and sorafenib were suppressed by DUXAP8 overexpression. Taken together, DUXAP8 gene silencing may be a potential strategy for improving the treatment effectiveness of sorafenib in HCC.

### 3.4 DUXAP8 sensitizes tumor cells to ferroptosis by regulating SLC7A11 level

**FIGURE 4:**
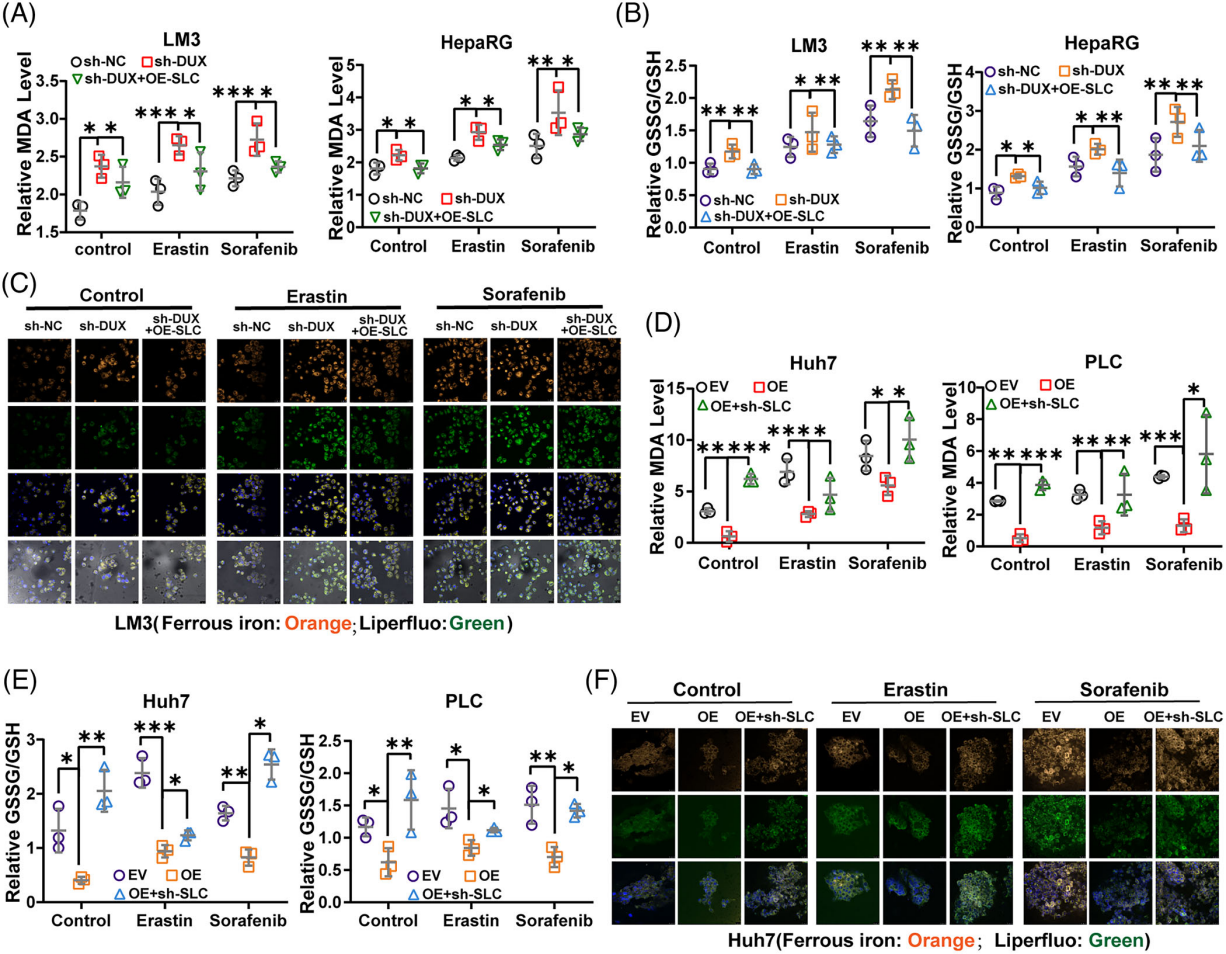
SLC7A11 mediates the regulatory effect of DUXAP8 on ferroptosis. (A, B) The DUXAP8 knockdown‐induced increases of intracellular MDA concentration and GSSG/GSH ratio level were reversed by SLC7A11 overexpression in both LM3 and HepaRG cells untreated and treated with erastin and sorafenib. (C) Representative IF images showed that the cellular lipid ROS (green) and Fe^2+^ (orange) levels were elevated by sh‐DUXAP8 transfection and the elevation could be reversed by SLC7A11 overexpression in LM3 cells untreated and treated with erastin and sorafenib. Magnification: ×400. (D, E) The DUXAP8 knockdown‐induced increases of intracellular MDA concentration and GSSG/GSH ratio level were reversed by SLC7A11 overexpression in both Huh7 and PLC cells untreated and treated with erastin and sorafenib. (F) Representative IF images showed that the cellular lipid ROS (green) and Fe^2+^ (orange) levels were elevated by sh‐DUXAP8 transfection and the elevations could be reversed by SLC7A11 overexpression in Huh7 cells untreated and treated with erastin and sorafenib. Magnification: ×400. * *p* < 0.05, ** *p* < 0.01, *** *p* < 0.001. NS, not significant. Results represent three independent experiments. sh‐DUX, sh‐DUXAP8; EV, Empty vector; OE, pcDNA DUXAP8; sh‐SLC, sh‐SLC7A11; OE‐SLC: pcDNA SLC7A11.

The effect of DUXAP8 on SLC7A11 protein levels could be reversed by SLC7A11 overexpression (Figure S5A,B) or knockdown (Figure S5C,D). We further manipulated SLC7A11 expression and explored the corresponding ferroptosis‐related changes in HCC cells. The results showed that DUXAP8 knockdown enhanced the promoting effects of erastin or sorafenib on lipid peroxidation and GSH depletion, and these effects could be blocked by SLC7A11 overexpression (Figure 4A,B). In addition, Liperfluo staining and the fluorescent probe FerroOrange showed that lipid ROS accumulation and intracellular Fe^2+^ level increased by sh‐DUXAP8 transfection was restored by SLC7A11 overexpression (Figures 4C and S5E). Consistently, the inhibitory effect of DUXAP8 overexpression on ferroptosis of both the MDA concentration and the GSSG/GSH ratio were reversed by SLC7A11 knockdown (Figure 4D,E). The lipid ROS accumulation and intracellular Fe^2+^ level decreased by DUXAP8 overexpression was restored by SLC7A11 knockdown (Figure 4F). These results further confirmed that SLC7A11 mediates the regulatory effect of DUXAP8 on ferroptosis.

### 3.5 DUXAP8 silencing reverses sorafenib resistance through enhancing ferroptosis

**FIGURE 5:**
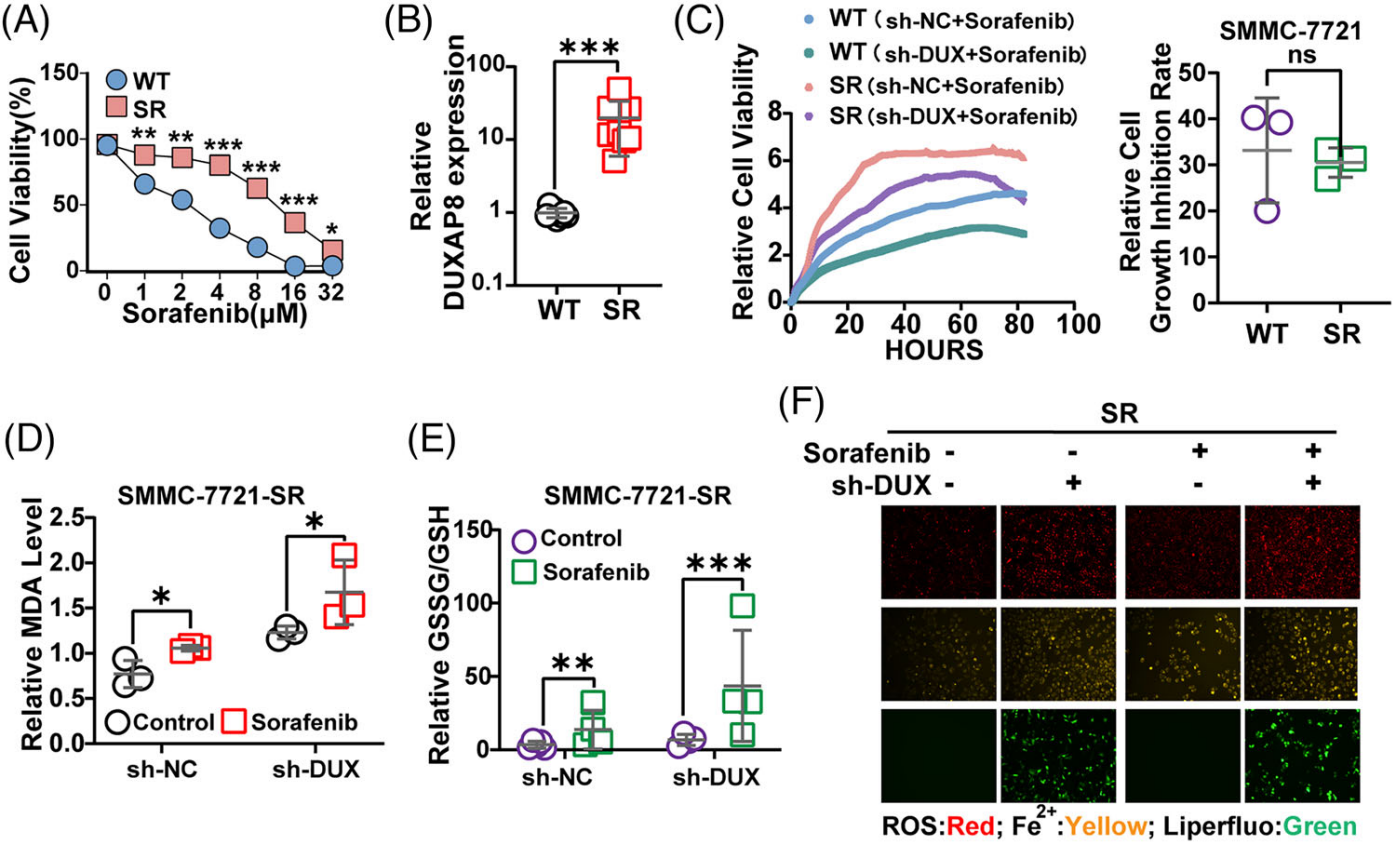
DUXAP8 is a key contributor to sorafenib resistance. To explore the role of DUXAP8 in sorafenib resistance of HCC cells, the HCC sorafenib‐resistant cell line SMMC‐7721‐SR (IC50 14.78 μg/mL) and sorafenib‐sensitive parental cell line SMMC‐7721‐WT (IC50 2.46 μg/mL) were used for in vitro tests. (A) Comparison of sorafenib resistance between SR and WT cells (CCK8 assay). (B) Comparison of DUXAP8 expression between SR and WT cells (qRT‐PCR assay). The statistical chart represents the comparison of cell growth inhibition rates of WT and SR cells after knockdown of DXUXAP8. (C) Inhibitory effect of DUXAP8 knockdown on the cell viability of both sorafenib‐treated wide‐type and sorafenib‐resistant cells measured by using the RTCA system. (D, E) DUXAP8 knockdown remarkably enhanced the changes of the MDA concentration (D) and GSSG/GSH ratio (E) induced by sorafenib (5 μM) in SMMC‐7721‐SR cells. (F) DUXAP8 knockdown enhanced the sorafenib (5 μM)—induced increases of ROS (red), ferrous iron (yellow), and intracellular lipid ROS (green) in sorafenib‐resistant cells. Magnification: ×200. * *p* < 0.05, ** *p* < 0.01, *** *p* < 0.001. Results represent three independent experiments. WT, SMMC‐7721‐WT; SR, SMMC‐7721‐SR; sh‐DUX, sh‐DUXAP8.

Compared with the SMMC‐7721‐WT (WT) cells, SMMC‐7721‐SR (sorafenib resistance,SR) cells were significantly less susceptible to sorafenib‐killing (Figure 5A). The DUXAP8 expression for sorafenib‐resistant (SR) cells was 14.68 times larger than that for WT cells (Figure 5B). Using the real‐time cell analyzer (RTCA) system, we found that silencing of DUXAP8 suppressed the growth of both sorafenib‐treated WT and SR cells. This suppression was not significantly different between WT and SR cells (Figure 5C). Also, DUXAP8 knockdown increased the proliferation, migration and invasion abilities of sorafenib‐treated SR cells (Figure S6A,B). Subsequently, a series of ferroptosis‐related parameters were tested. Knockdown of DUXAP8 appeared to increase MDA production (Figure 5D) and GSSG/GSH (Figure 5E) more markedly in SR cells treated with sorafenib than in cells without sorafenib treatment. Total ROS and lipid ROS, as well as accumulation of ferrous iron, verified that DUXAP8 knockdown could remarkably increase ferroptosis in the SR cell lines (Figure 5F). The results above confirmed that DUXAP8 may contribute to sorafenib resistance and silencing DUXAP8 may enhance tumor cell ferroptosis.

### 3.6 DUXAP8 binds to SLC7A11 protein and promotes SLC7A11 lysosomal sorting

**FIGURE 6:**
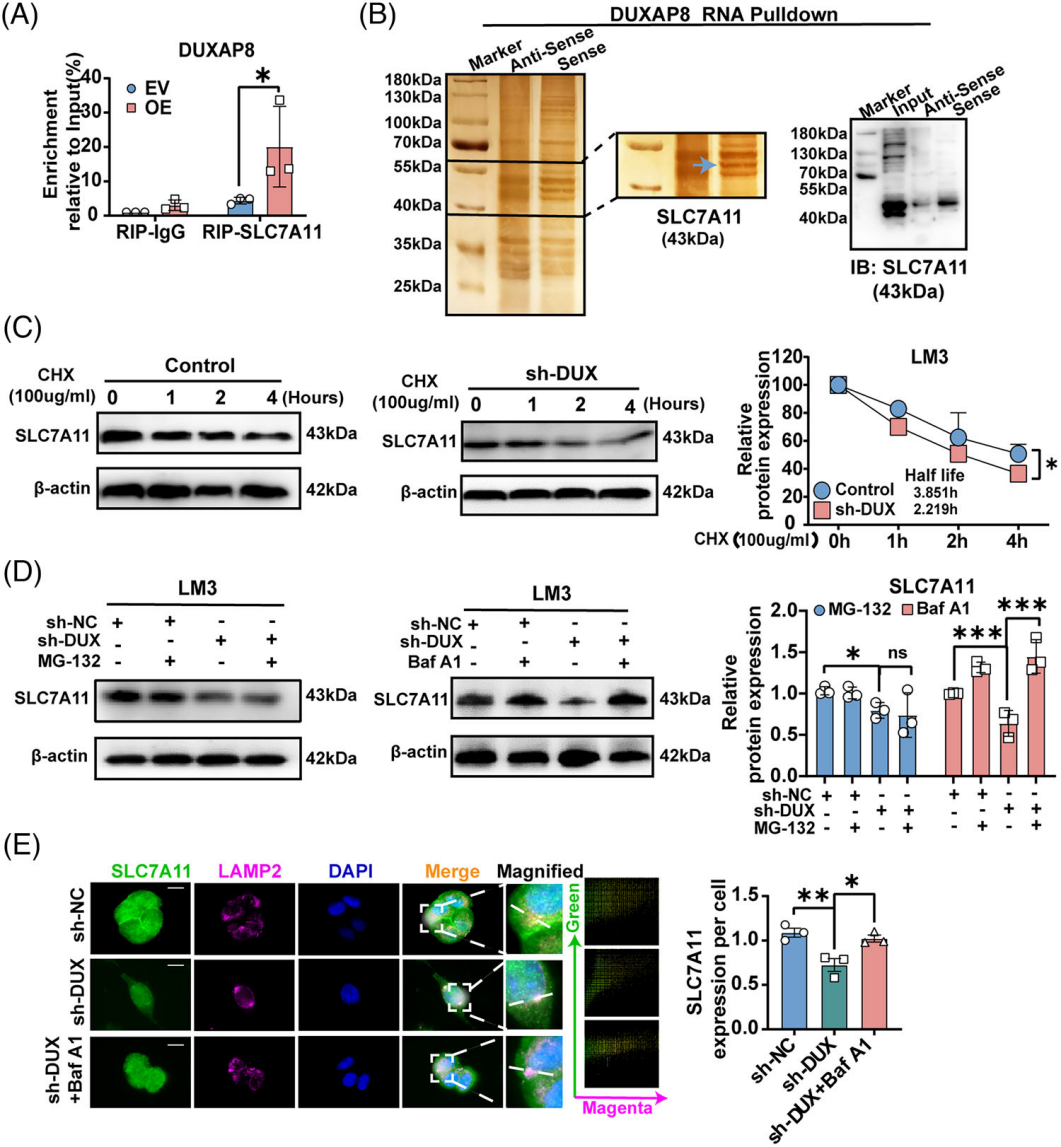
LncRNA DUXAP8 directly binds to SLC7A11 and promotes lysosomal sorting. (A) Binding of SLC7A11 and DUXAP8 detected by RIP assay. (B) SLC7A11 was abundantly enriched in biotinylated DUXAP8 precipitates as detected by silver staining and WB assay. (C) Half‐life of SLC7A11 protein in LM3 cells with and without DUXAP8 knockdown. Cycloheximide (CHX), 100 mg/mL. (D) Effects of MG‐132 (20 μM) and bafilomycin A1 (Baf A1, 10 μM) treatment for 6 h on SLC7A11 expression in LM3 cells with and without DUXAP8 knockdown. (E) DUXAP8 knockdown reduced the distribution of SLC7A11 (green) in lysosomes (marked by LAMP2, magenta). The scatter diagram was conducted by using the Image Pro Plus software. * *p* < 0.05, **p < 0.01, ***p < 0.001. NS, not significant. Results represent three independent experiments. sh‐DUX, sh‐DUXAP8; EV, Empty vector; OE, pcDNA DUXAP8.

Further, the molecular mechanism underlying the effect of DUXAP8 on SLC7A11 protein was investigated. RNA immunoprecipitation revealed a significant binding of SLC7A11 and DUXAP8 in LM3 cells (Figure 6A). RNA pull‐down assay showed that SLC7A11 was abundantly enriched in biotinylated DUXAP8 precipitates (Figure 6B). In addition, by blocking protein synthesis with CHX, we addressed that DUXAP8 knockdown shortened the half‐life of SLC7A11 in LM3 cells (Figure 6C). For degradation, membrane proteins are transported to lysosomes and/or proteasomes. In LM3 cells, sh‐DUXAP8‐enhanced SLC7A11 degradation was reduced by the lysosome inhibitor Baf A1 but unchanged by the proteasome inhibitor MG‐132 (Figure 6D). Immunofluorescence co‐staining demonstrated that the distribution of SLC7A11 in lysosomes significantly increased in the sh‐DUXAP8 group, which could be rescued by Baf A1 (Figure 6E). Also, an assessment of lysosome‐related genes associated with DUXAP8 was conducted, and found that 30 genes were positively correlated with DXAUP8 and 3 genes were negatively correlated with DXUAP8, which confirms that DUXAP8 may be an interacting lncRNA with lysosome (Table S2). These results suggest that DUXAP8 may interact with SLC7A11 and regulate its stability predominantly through lysosomal pathway.

### 3.7 DUXAP8 increases the palmitoylation of SLC7A11 protein

**FIGURE 7:**
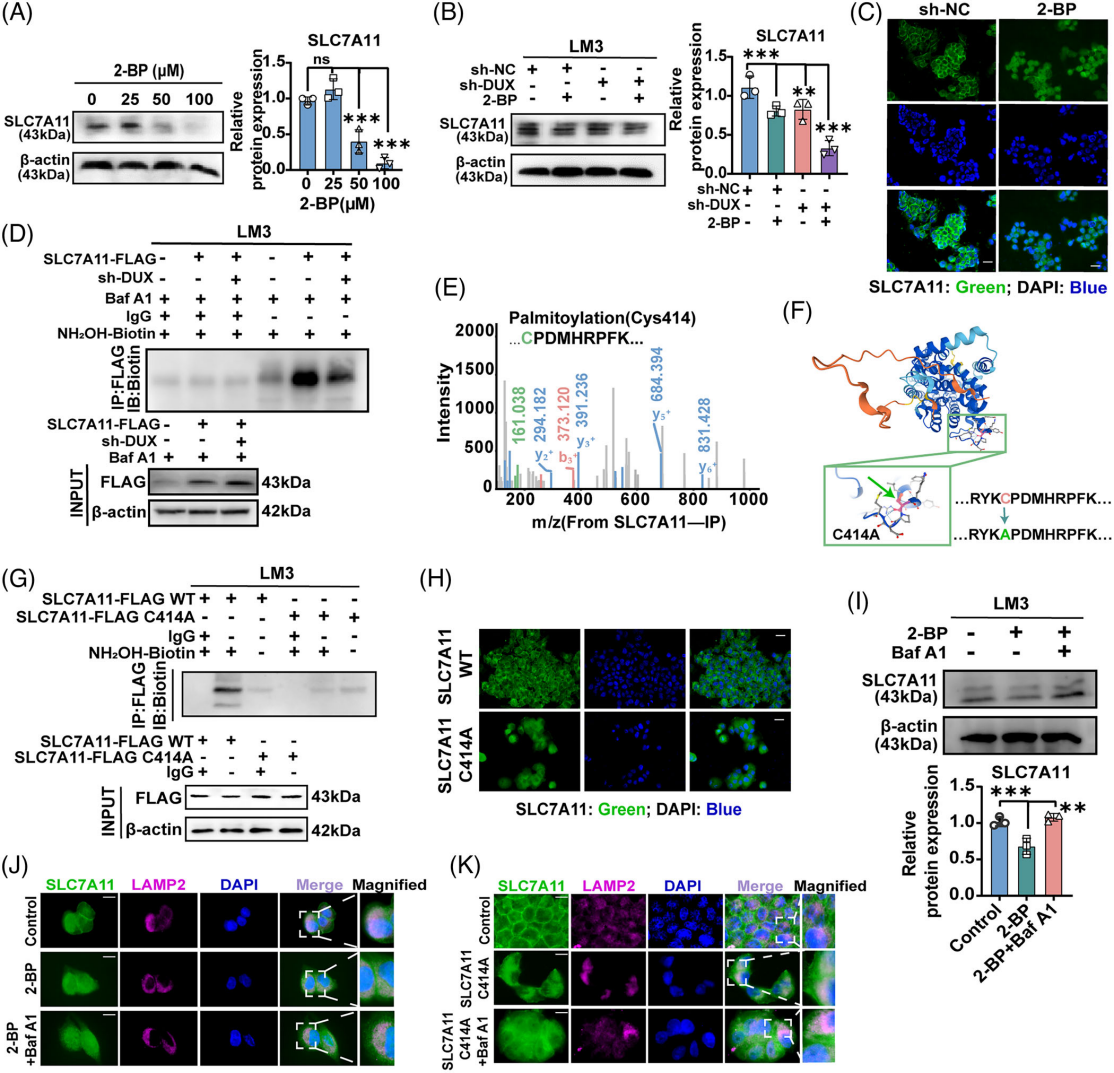
DUXAP8 promotes SLC7A11 palmitoylation and lysosomal sorting. (A) SLC7A11 expression was decreased by 2‐BP in a dose‐dependent manner in LM3 cells. The cells were treated for 24 h. (B) 2‐BP (50 μM) significantly decreased SLC7A11 expression in LM3 cells, especially the cells with DUXAP8 knockdown. (C) Visualization (IF staining) of SLC7A11 (green) in LM3 cells treated with 2‐BP (50 μM) for 24 h. Magnification: ×400. (D) DUXAP8 knockdown weakened the palmitoylation of exogenous SLC7A11 in LM3 cells, which were pretreated with 50 μM Baf A1 for 2 h. Lysates were incubated with antibodies against Flag and biotin‐tagged NH2OH for immunoprecipitation and immunoblotting, respectively. (E) The single palmitoylation site of SLC7A11 at Cys414 was identified by the mass spectrum. (F) The alphafold2 database predicted the protein structure of human SLC7A11. (G) LM3 cells overexpressing SLC7A11‐Flag or the C414A‐Flag mutant were cultured with or without NH2OH were then prepared for the ABE method and then prepared for ABE reaction. The palmitoylated proteins were precipitated using streptavidin bead conjugate and blotted with Flag and streptavidin HRP antibodies. (H) After transfection of SLC7A11 or C411A mutant, LM3 cells were immunostained for SLC7A11 (green). Magnification: ×400. (I) Expression of SLC7A11 in LM3 cells pretreated with 2‐BP or Baf A1. (J) Representative IF images were showing the co‐localization between SLC7A11 (green) and LAMP2 (magenta) incubated with or without 2‐BP/ Baf A1. (K) Representative IF images showed the effect of C414A mutation on tumor SLC7A11 lysosomal localization. LM3 cells were ectopically expressed with SLC7A11 or C414A mutants stained for SLC7A11 (green) and LAMP2 (magenta). The cell nucleus (blue) was stained with DAPI. *p < 0.05, **p < 0.01, ***p < 0.001. NS, not significant. Results represent three independent experiments. sh‐DUX, sh‐DUXAP8; IP, immunoprecipitation; IB, immunoblotting.

Translocation of SLC7A11 onto the plasma membrane is a crucial step in ferroptosis,^28^, ^29^ while palmitoylation was important in regulating protein localization and lysosome sorting.^30^ Therefore, we explored whether DUXAP8 facilitates SLC7A11 palmitoylation and lysosomal sorting. The results showed that 2‐bromopalmitate (2‐BP), a palmitoylation inhibitor, decreased the expression of SLC7A11 in a dose‐dependent manner (Figures 7A and S7A). 2‐BP in combination with sh‐DUXAP8 transfection could further reduce SLC7A11 expression (Figure 7B). The effects of 2‐BP on SLC7A11 localization and expression were also visualized and confirmed by immunofluorescence staining (Figure 7C). Acyl biotin exchange (ABE) assay detected potent SLC7A11 palmitoylation (Figure S7B). ABE assay demonstrated that DUXAP8 attenuated the palmitoylation of SLC7A11 in LM3 and 293T cells (Figures 7D and S7C,D). Further, the predictor CSS‐palm 4.0 and Swiss‐Plam both consistently predicted six palmitoylation sites of SLC7A11 within its cysteine residues (Figure S7E,F). SLC7A11 was then subjected to mass spectrometric analysis and the results confirmed that Cys414 is a single conservative palmitoylation site (Figure 7E). SLC7A11 palmitoylation was utterly abolished by substituting the Cys414 residue with alanine (C414A) (Figures 7F,G and S7G). The C414A mutation decreased membrane distribution and expression of SLC7A11 protein (Figure 7H). The 2‐BP‐induced alleviation of SLC7A11 protein content was increased by the Baf A1 (Figure 7I). Next, 2‐BP treatment and C414A mutation resulted in an increase in lysosomal SLC7A11 and disrupted SLC7A11 protein stability in LM3 cells, while Baf A1 treatment showed opposite effects and reduced SLC7A11 in lysosomes (Figure 7J,K). The findings suggest that DUXAP8 can enhance palmitoylation of SLC7A11, in which Cys414 plays a predominant role, and suppress SLC7A11 degradation through the lysosomal pathway.

### 3.8 Silencing DUXAP8 inhibits tumorigenesis of HCC cells and improves sorafenib efficacy in vivo

**FIGURE 8:**
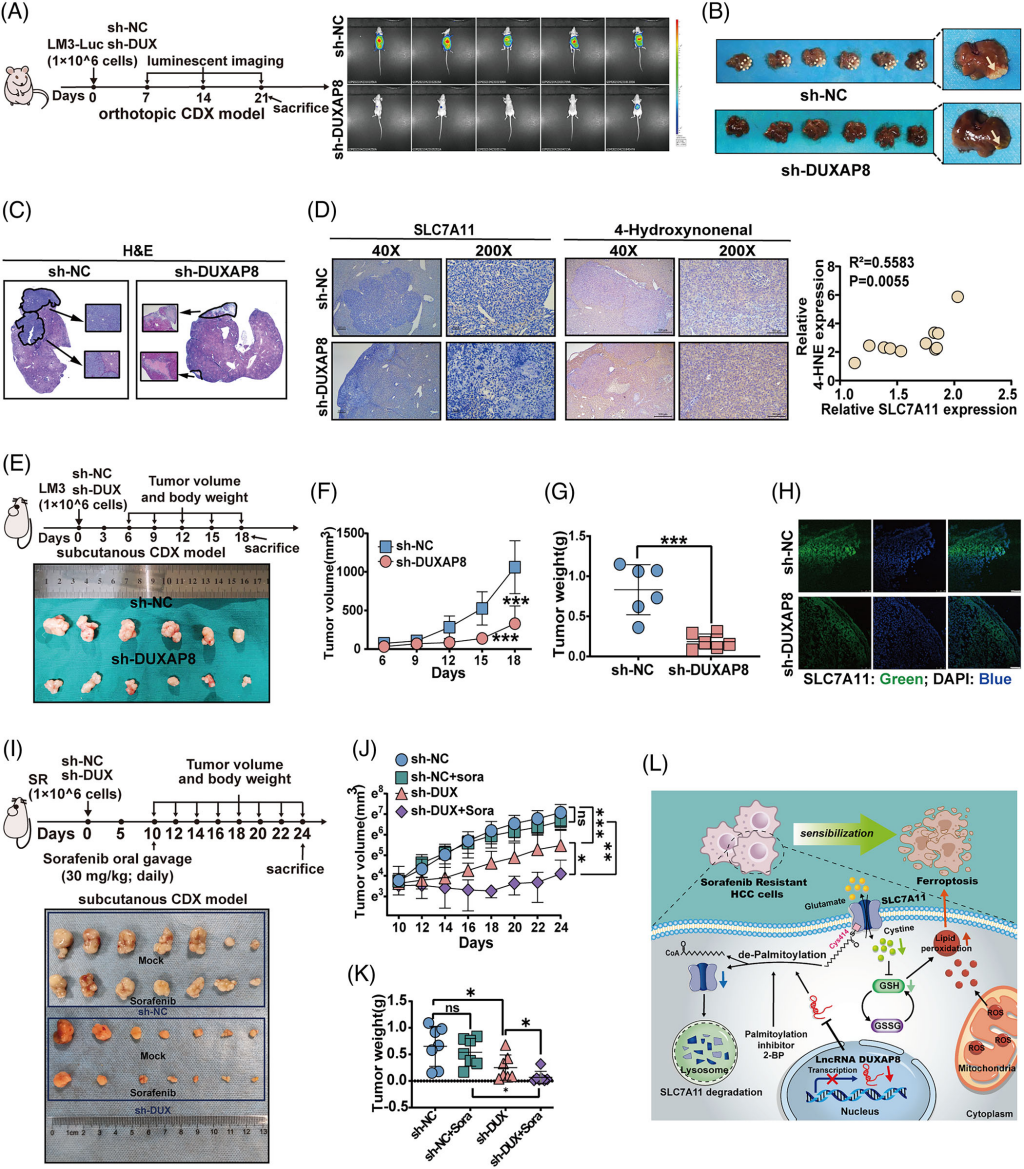
Silencing DUXAP8 exerts its antitumor effects in vivo. (A) BALB/c nude mice were orthotopically implanted with LM3‐Luc‐shNC or LM3‐Luc‐shDUXAP8 cells. In vivo luminescent imaging was performed weekly starting on day 7. Animals were sacrificed on day 21. Luminescence intensity ranges from low (blue) to high (red). (B) Representative photograph of the orthotopic tumor of LM3 cells in mice liver. (C) Comparison of histological features of HCC orthotopic CDX tissues. Magnification: ×40 (big) and ×200 (small). (D) IHC for SLC7A11 and 4‐hydroxynonenal in sh‐DUXAP8 and sh‐NC LM3 xenografts. Magnification: ×40 (top panel) and ×200 (bottom panel). Quantification of the IHC staining showed the correlation of SLC7A11 and 4‐HNE expression (N = 10). Statistical significance was calculated using Pearson correlation analysis. (E) BALB/c nude mice were subcutaneous injected with LM3‐NC or LM3‐shDUXAP8 cells. Representative photograph of the subcutaneous tumor of LM3 cells. (F) Tumor volume was measured every three days starting from Day 6. (G) Tumor weight of the two groups of mice. (H) Representative IF images of xenograft tumors for SLC7A11 expression (green). Magnification: ×400. (I) BALB/c nude mice were subcutaneous injected with SR‐shNC or SR‐shDUXAP8 cells. Sorafenib or castor oil was administered by gavage every day from day 10. Representative photograph of the subcutaneous tumor of LM3 cells. (J) Tumor volume was measured every two days starting from Day 10. (K) Tumor weight of the four groups of mice. (L) Proposed mechanistic depiction for DUXA8 mediated ferroptosis in HCC sorafenib resistance. *p < 0.05, **p < 0.01, ***p < 0.001. NS, not significant. sh‐DUX, sh‐DUXAP8.

To further evaluate the bio‐functional effect of DUXAP8 on tumorigenesis in vivo, we constructed three mouse models. In the orthotopic‐CDX mouse models, the liver tumor mass grew remarkably slower in the mice of the sh‐DUXAP8 group when compared with those in the sh‐NC group (Figures 8A and S8A), and the number and size of tumors remarkably decreased (Figure 8B,C). IHC testing revealed that SLC7A11 expression decreased and lipid peroxide 4‐hydroxynonenal (4‐HNE) increased in the tumor samples of the sh‐DUXAP8 group, and a positive correlation was observed between SLC7A11 and 4‐HNE levels (Figure 8D). In addition, multiple lung metastatic lesions of primary HCC tumors were found in the control group (Figure S8B). Similar results were observed in the subcutaneous CDX models. The mice in the sh‐DUXAP8 group had significantly slower increases of tumor volume and weight (Figure 8E–G) but did not show a statistically difference in the body weight (Figure S8C). Tumors formed from sh‐DUXAP8‐transfected cells exhibited lower fluorescence intensity for SLC7A11 (Figures 8H and S8D). In the SR CDX model, the tumor volume and weight confirmed that the sensitivity of mice with implantation of SR cells expressing sh‐DUXAP8 to sorafenib was markedly higher (Figure 8I–K). The general condition of mice, assessed based on body weight, liver and kidney functions, did not demonstrate significant differences (Figure S8E,F). Others, as shown in the representative image in Figure S8G, silent DUXAP8 had little effect on cell proliferation, apoptosis or necrosis in orthotopic and subcutaneous transplanted tumors in mice. Depriving DUXAP8 can suppress tumor growth and improve sorafenib efficacy in clinical HCC patients.

In summary, lncRNA DUXAP8 may be used in combination with sorafenib to achieve a higher anti‐liver cancer efficacy by acting on the SLC7A11‐mediated ferroptosis pathway. DUXAP8 regulates the palmitoylation level of SLC7A11 and protein stability (Figure 8L).

## 4 DISCUSSION

Treatment options for advanced HCC are still very limited, such as molecular targeted therapy, often rapidly trigger compensatory drug‐resistance mechanisms that limit their efficacy.^31^, ^32^, ^33^ The clinical benefit of sorafenib has mainly been limited by drug resistance developing in patients with HCC.^15^, ^34^ The lethality of sorafenib in HCC treatment is related to system xc^−^ inhibition and ferroptosis.^35^ Thus, the focus of this study was on understanding how to enhance the ferroptosis induced by sorafenib and suppress sorafenib resistance.

In the present study, we found that the expression level of DUXAP8 in HCC tumor tissue was significantly higher than in adjacent benign tissue using data from TCGA database and the tissue samples of HCC patients diagnosed at our hospital. A high expression level of DUXAP8 in HCC tissues was significantly associated with a worse clinical stage and poor prognosis. In vitro, we showed that DUXAP8 expression enhanced tumor cell proliferation, migration, and invasion, and promoted HCC progression. Consistent with published reports,^20^, ^21^, ^36^ DUXAP8 is critical for assessing prognosis which may act as an oncogenic lncRNA in HCC.

Ferroptosis modulates the pathogenesis and the progression of HCC. For example, metallothionein‐1G,^15^ the p62/NRF2 pathway,^26^ and the S47 variant of the TP53 gene^37^ all protect against ferroptosis and hepatocarcinogenesis.^38^, ^39^, ^40^ However, the regulation of SLC7A11 and cell ferroptosis by lncRNA remains largely uncharacterized. Here, we report that DUXAP8 induced cystine depletion, substantial enhancement of lipid peroxidation, and ferrous ion metabolic disorder in HCC cell lines, while SLC7A11 could reverse the effects of DUXAP8 on ferroptosis in HCC. Further, h‐DUXAP8 alleviates sorafenib resistance and improve sorafenib efficacy in HCC cells. Targeting DUXAP8 can be successfully combined with strategies to induce ferroptosis with sorafenib, if generalizable, it creates an effective strike against HCC resistant cells.

Previous studies had shown that the cytomembrane localized SLC7A11 increases cystine uptake and glutamate production, consequently mediating cell ferroptosis. Through promoting protein binding to the cell membrane,^41^ palmitoylation is a pivotal mechanism regulating the trafficking and functions of multiple tumor‐related proteins, including PD‐L1, STAT3, and IFNGR1.^30^, ^42^, ^43^ The solute carrier (SLC) transporters had been previously shown to be substrates for palmitoylation.^44^, ^45^ Hence, we first explored the role of palmitoylation in the effect of DUAXP8 on SLC7A11. We found that DUXAP8 could bind to SLC7A11, DUXAP8 knockdown impeded SLC7A11 palmitoylation and prompted sorting to the lysosome for degradation. In addition, SLC7A11 was palmitoylated on Cys414 and DUXAP8 contributed to SLC7A11 palmitoylation at Cys414 to maintain cytomembrane localization of SLC7A11. This phenomenon extended the functional layers of SLC7A11 regulated by post‐translational modifications, and highlighted the importance of lncRNA in cancer metabolic reprogramming and ferroptosis mediated by SLC7A11.

However, there are certain drawbacks to this study. The mechanism of DUXAP8 enhancing palmitoylation of SLC7A11 was unclear. Further experiments are still needed to explore whether DUXAP8 is associated with a potential lysosomal factor to promote palmitoylation of SLC7A11, and the binding site and binding mode of DUXAP8 with lysosomal gene. In fact, lncRNAs are not widely used in clinical applications at present. The vast majority of lncRNAs are used in clinical disease screening and diagnosis by means of gene arrays. Notably, siRNAs, delivered by nanomaterials, lipid mediators and exosomes, can specifically silence disease related genes and are being explored as anti‐cancer agents at a pre‐clinical or clinical stage.^41^, ^46^ Next, targeting lncRNA DUXAP8 via a siRNA interference approach, might be an efficient treatment in clinics. Thus, our findings provide considerable insight into a new strategy for improving clinical prognosis and neoadjuvant treatment of HCC.

In summary, lncRNA DUXAP8 contributes to the malignancies of HCC by inhibiting SLC7A11 de‐palmitoylation and reducing its degradation to suppress ferroptosis. In addition, DUXAP8 silencing in combination with sorafenib may improve treatment efficacy in patients with advanced HCC.

## 5 CONCLUSION

In summary, lncRNA DUXAP8 contributes to the malignancies of HCC by inhibiting SLC7A11 de‐palmitoylation and reducing its degradation to suppress ferroptosis. In addition, DUXAP8 silencing in combination with sorafenib may improve treatment efficacy in patients with advanced HCC.

## CONFLICTS OF INTEREST STATEMENT

The authors declare that they have no conflicts of interest.

## Supporting information

Supporting Information

## References

[1] Sung H , Ferlay J , Siegel RL , et al. Global cancer statistics 2020: gLOBOCAN estimates of incidence and mortality worldwide for 36 cancers in 185 countries. CA: Cancer J Clin. 2021;71(3):209‐249. 33538338 10.3322/caac.21660

[2] Jin H , Shi Y , Lv Y , et al. EGFR activation limits the response of liver cancer to lenvatinib. Nature. 2021;595(7869):730‐734. 34290403 10.1038/s41586-021-03741-7

[3] Cucchetti A , Zhong J , Berhane S , et al. The chances of hepatic resection curing hepatocellular carcinoma. J Hepatol. 2020;72(4):711‐717. 31790765 10.1016/j.jhep.2019.11.016

[4] Yu S , Ma C , Heinrich B , et al. Targeting the crosstalk between cytokine‐induced killer cells and myeloid‐derived suppressor cells in hepatocellular carcinoma. J Hepatol. 2019;70(3):449‐457. 30414862 10.1016/j.jhep.2018.10.040 PMC6380944

[5] Ricke J , Schinner R , Seidensticker M , et al. Liver function after combined selective internal radiation therapy or sorafenib monotherapy in advanced hepatocellular carcinoma. J Hepatol. 2021;75(6):1387‐1396. 34454995 10.1016/j.jhep.2021.07.037

[6] Kolamunnage‐Dona R , Berhane S , Potts H , et al. Sorafenib is associated with a reduced rate of tumour growth and liver function deterioration in HCV‐induced hepatocellular carcinoma. J Hepatol. 2021;75(4):879‐887. 34052255 10.1016/j.jhep.2021.05.015 PMC9158473

[7] Ma M , Lau E , Leung D , et al. Stearoyl‐CoA desaturase regulates sorafenib resistance via modulation of ER stress‐induced differentiation. J Hepatol. 2017;67(5):979‐990. 28647567 10.1016/j.jhep.2017.06.015

[8] Wu F , Fang T , Yu L , et al. ADRB2 signaling promotes HCC progression and sorafenib resistance by inhibiting autophagic degradation of HIF1α. J Hepatol. 2016;65(2):314‐324. 27154061 10.1016/j.jhep.2016.04.019

[9] Wang H , Zhang S , Zhang Y , et al. TAZ is indispensable for c‐MYC‐induced hepatocarcinogenesis. J Hepatol. 2021;76(1):123‐134. 34464659 10.1016/j.jhep.2021.08.021 PMC9569156

[10] Wang Q , Liang N , Yang T , et al. DNMT1‐mediated methylation of BEX1 regulates stemness and tumorigenicity in liver cancer. J Hepatol. 2021;75(5):1142‐1153. 34217777 10.1016/j.jhep.2021.06.025

[11] Zhang Y , Shi J , Liu X , et al. BAP1 links metabolic regulation of ferroptosis to tumour suppression. Nat Cell Biol. 2018;20(10):1181‐1192. 30202049 10.1038/s41556-018-0178-0 PMC6170713

[12] Doll S , Freitas F , Shah R , et al. FSP1 is a glutathione‐independent ferroptosis suppressor. Nature. 2019;575(7784):693‐698. 31634899 10.1038/s41586-019-1707-0

[13] Bansal A , Simon M . Glutathione metabolism in cancer progression and treatment resistance. J Cell Biol. 2018;217(7):2291‐2298. 29915025 10.1083/jcb.201804161 PMC6028537

[14] Fang X , Cai Z , Wang H , et al. Loss of cardiac ferritin H facilitates cardiomyopathy via Slc7a11‐mediated ferroptosis. Circ Res. 2020;127(4):486‐501. 32349646 10.1161/CIRCRESAHA.120.316509

[15] Sun X , Niu X , Chen R , et al. Metallothionein‐1G facilitates sorafenib resistance through inhibition of ferroptosis. Hepatology. 2016;64(2):488‐500. 27015352 10.1002/hep.28574 PMC4956496

[16] Dolma S , Lessnick S , Hahn W , Stockwell B . Identification of genotype‐selective antitumor agents using synthetic lethal chemical screening in engineered human tumor cells. Cancer Cell. 2003;3(3):285‐296. 12676586 10.1016/s1535-6108(03)00050-3

[17] Li R , Tian T , Ge Q , et al. A phosphatidic acid‐binding lncRNA SNHG9 facilitates LATS1 liquid‐liquid phase separation to promote oncogenic YAP signaling. Cell Res. 2021;31(10):1088‐1105. 34267352 10.1038/s41422-021-00530-9 PMC8486796

[18] Li G , Kryczek I , Nam J , et al. LIMIT is an immunogenic lncRNA in cancer immunity and immunotherapy. Nat Cell Biol. 2021;23(5):526‐537. 33958760 10.1038/s41556-021-00672-3 PMC8122078

[19] Li Z , Hou P , Fan D , et al. The degradation of EZH2 mediated by lncRNA ANCR attenuated the invasion and metastasis of breast cancer. Cell Death Differ. 2017;24(1):59‐71. 27716745 10.1038/cdd.2016.95 PMC5260507

[20] Lian Y , Yang J , Lian Y , Xiao C , Hu X , Xu H . DUXAP8, a pseudogene derived lncRNA, promotes growth of pancreatic carcinoma cells by epigenetically silencing CDKN1A and KLF2. Cancer Commun. 2018;38(1):64. 10.1186/s40880-018-0333-9 PMC6235391 30367681

[21] Sun M , Nie F , Zang C , et al. The pseudogene DUXAP8 promotes non‐small‐cell lung cancer cell proliferation and invasion by epigenetically silencing EGR1 and RHOB. Mol Ther. 2017;25(3):739‐751. 28131418 10.1016/j.ymthe.2016.12.018 PMC5363203

[22] Hu Y , Zhang X , Zai H , Jiang W , Xiao L , Zhu Q . lncRNA DUXAP8 facilitates multiple malignant phenotypes and resistance to PARP inhibitor in HCC via upregulating FOXM1. Mol Ther Oncolytics. 2020;19:308‐322. 33313387 10.1016/j.omto.2020.10.010 PMC7701012

[23] Wu S , Zhang L , Deng J , et al. A novel micropeptide encoded by Y‐linked LINC00278 links cigarette smoking and AR signaling in male esophageal squamous cell carcinoma. Cancer Res. 2020;80(13):2790‐2803. 32169859 10.1158/0008-5472.CAN-19-3440

[24] Mukhopadhyay S , Biancur D , Parker S , et al. Autophagy is required for proper cysteine homeostasis in pancreatic cancer through regulation of SLC7A11. Proc Natl Acad Sci USA. 2021;118(6):e2021475118. 33531365 10.1073/pnas.2021475118 PMC8017731

[25] Dixon S , Patel D , Welsch M , et al. Pharmacological inhibition of cystine‐glutamate exchange induces endoplasmic reticulum stress and ferroptosis. Elife. 2014;3:e02523. 24844246 10.7554/eLife.02523 PMC4054777

[26] Sun X , Ou Z , Chen R , et al. Activation of the p62‐Keap1‐NRF2 pathway protects against ferroptosis in hepatocellular carcinoma cells. Hepatology. 2016;63(1):173‐184. 26403645 10.1002/hep.28251 PMC4688087

[27] Luo M , Wu L , Zhang K , et al. miR‐137 regulates ferroptosis by targeting glutamine transporter SLC1A5 in melanoma. Cell Death Differ. 2018;25(8):1457‐1472. 29348676 10.1038/s41418-017-0053-8 PMC6113319

[28] Liu T , Jiang L , Tavana O , Gu W . The deubiquitylase OTUB1 mediates ferroptosis via stabilization of SLC7A11. Cancer Res. 2019;79(8):1913‐1924. 30709928 10.1158/0008-5472.CAN-18-3037 PMC6467774

[29] Wang W , Green M , Choi J , et al. CD8 T cells regulate tumour ferroptosis during cancer immunotherapy. Nature. 2019;569(7755):270‐274. 31043744 10.1038/s41586-019-1170-y PMC6533917

[30] Yao H , Lan J , Li C , et al. Inhibiting PD‐L1 palmitoylation enhances T‐cell immune responses against tumours. Nat Biomed Eng. 2019;3(4):306‐317. 30952982 10.1038/s41551-019-0375-6

[31] Xu J , Ji L , Liang Y , et al. CircRNA‐SORE mediates sorafenib resistance in hepatocellular carcinoma by stabilizing YBX1. Signal Transduct Target Ther. 2020;5(1):298. 33361760 10.1038/s41392-020-00375-5 PMC7762756

[32] Llovet J , Ricci S , Mazzaferro V , et al. Sorafenib in advanced hepatocellular carcinoma. N Engl J Med. 2008;359(4):378‐390. 18650514 10.1056/NEJMoa0708857

[33] Lencioni R , Llovet J , Han G , et al. Sorafenib or placebo plus TACE with doxorubicin‐eluting beads for intermediate stage HCC: the SPACE trial. J Hepatol. 2016;64(5):1090‐1098. 26809111 10.1016/j.jhep.2016.01.012

[34] Zhang P , Li K , Shen Y , et al. Galectin‐1 induces hepatocellular carcinoma EMT and sorafenib resistance by activating FAK/PI3K/AKT signaling. Cell Death Dis. 2016;7:e2201. 27100895 10.1038/cddis.2015.324 PMC4855644

[35] Chu B , Kon N , Chen D , et al. ALOX12 is required for p53‐mediated tumour suppression through a distinct ferroptosis pathway. Nat Cell Biol. 2019;21(5):579‐591. 30962574 10.1038/s41556-019-0305-6 PMC6624840

[36] Huang T , Wang X , Yang X , et al. Long non‐coding RNA DUXAP8 enhances renal cell carcinoma progression via downregulating miR‐126. Med Sci Monit. 2018;24:7340‐7347. 30317248 10.12659/MSM.910054 PMC6198709

[37] Jennis M , Kung C , Basu S , et al. An African‐specific polymorphism in the TP53 gene impairs p53 tumor suppressor function in a mouse model. Genes Dev. 2016;30(8):918‐930. 27034505 10.1101/gad.275891.115 PMC4840298

[38] Wang W , Green M , Choi JE , et al. CD8+ T cells regulate tumour ferroptosis during cancer immunotherapy. Nature. 2019;569(7755):270‐274. 31043744 10.1038/s41586-019-1170-y PMC6533917

[39] Lin C , Lin P , Lin C , et al. Decreased mRNA expression for the two subunits of system xc(‐), SLC3A2 and SLC7A11, in WBC in patients with schizophrenia: evidence in support of the hypo‐glutamatergic hypothesis of schizophrenia. J Psychiatr Res. 2016;72:58‐63. 26540405 10.1016/j.jpsychires.2015.10.007

[40] Li Y , Li J , Li Z , et al. Homeostasis imbalance of YY2 and YY1 promotes tumor growth by manipulating ferroptosis. Adv Sci. 2022;9(13):e2104836. 10.1002/advs.202104836 PMC9069185 35246964

[41] Butt AM , Amin MCIM , Katas H , Abdul Murad NA , Jamal R , Kesharwani P . Doxorubicin and siRNA codelivery via chitosan‐coated pH‐Responsive mixed micellar polyplexes for enhanced cancer therapy in multidrug‐resistant tumors. Mol Pharmaceut. 2016;13(12):4179‐4190. 10.1021/acs.molpharmaceut.6b00776 27934479

[42] Zhang M , Zhou L , Xu Y , Yang M , Xu Y , Komaniecki G , et al. A STAT3 palmitoylation cycle promotes T17 differentiation and colitis. Nature. 2020;586(7829):434‐439. 33029007 10.1038/s41586-020-2799-2 PMC7874492

[43] Du W , Hua F , Li X , et al. Loss of optineurin drives cancer immune evasion via palmitoylation‐dependent IFNGR1 lysosomal sorting and degradation. Cancer Discov. 2021;11(7):1826‐1843. 33627378 10.1158/2159-8290.CD-20-1571 PMC8292167

[44] Rastedt DE , Vaughan RA , Foster JD . Palmitoylation mechanisms in dopamine transporter regulation. J Chem Neuroanat. 2017;83‐84:3‐9. 10.1016/j.jchemneu.2017.01.002 PMC6077000 28115272

[45] Hilgemann DW , Fine M , Linder ME , Jennings BC , Lin M‐J . Massive endocytosis triggered by surface membrane palmitoylation under mitochondrial control in BHK fibroblasts. Elife. 2013;2:e01293. 24282236 10.7554/eLife.01293 PMC3839538

[46] Kim H , Jeong IH , Choi YK , et al. Suppression of lung cancer malignancy by micellized siRNA through cell cycle arrest. Adv Healthc Mater. 2023:e2202358. 36644959 10.1002/adhm.202202358

