# Supplementary material for: Loss of LncRNA DUXAP8 synergistically enhanced sorafenib induced ferroptosis in hepatocellular carcinoma via SLC7A11 de‐palmitoylation
Source: Clin Transl Med. 2023 Jun 19;13(6):e1300. doi: 10.1002/ctm2.1300 (PMC10280000; doi:10.1002/ctm2.1300)
Supplement: Supplementary file 1 — Supporting Information [file CTM2-13-e1300-s001.docx]

**Supplementary Methods**

**Microarray analysis and TCGA Dataset Processing**

RNA-sequencing (RNA-Seq) data of TCGA-LIHC were downloaded from the TCGA database (https://portal.gdc.cancer.gov/), and corresponding clinical characteristics were obtained from cBioPortal (https://www.cbioportal.org/). The RNA-seq data (FPKM values) were normalized to log2 (FPKM+1). The differential expression of DUXAP8 was investigated between 374 tumors and 50 normal tissues using the Wilcoxon test and 50 paired tissues utilizing Wilcoxon paired test, respectively. X-tile software [^46^](#_ENREF_46) and Kaplan-Meier (K-M) survival curves with the log-rank test were generated to investigate the difference in the overall survival (OS) and disease-free survival (DFS) based on the DUXAP8 expression level (patients with missing survival information were excluded). Patients with complete clinical information were incorporated into the univariate and multivariate Cox regression analyses. The expression of ferroptosis key genes from 374 HCC patients has evaluated their co-expression relationship with DUXAP8 by Pearson correlation analysis. Gene set enrichment analysis (GSEA) was applied to assess three sets of genes downloaded from the MSigDB database (http://www.gsea-msigdb.org/) group by the median DUXAP8 expression level using the OmicStudio tools at https://www.omicstudio.cn/tool, including GOMF_IRON_ION_BINDING, GOBP_LIPID_METABOLIC_PROCESS, and GOBP_REACTIVE_OXYGEN_SPECIES_BIOS

YNTHETIC_PROCESS. The complete clinical information is listed in Table S1.

**Cell culture**

were obtained from the Academy of Sciences Cell Bank of China.SMMC-7721 wild type and SMMC-7721 sorafenib resistance cell lines were acquired from the Bena culture collection. All cell lines were authenticated by short tandem repeat DNA profiling. These cells were grown in Roswell Park Memorial Institute (RPMI) 1640 Medium (HepaRG, Li-7, SUN-387, SUN-182 and L02) or Dulbecco’s modified Eagle’s medium (HuH7, LM3, Hep3B, PLC, LX2, 293T and SMMC-7721) with 10% fetal bovine serum and 100 U/mL penicillin and streptomycin. We choose the dosage according to the IC50 of all the drugs in the corresponding cells.

**Plasmid and Lentivirus transfection**

All shRNAs (DUXAP8) and pcDNAs (DUXAP8 and SLC7A11) were purchased from genechem. pcDNA SLC7A11-Flag was from Public Protein/Plasmid Library, and pcDNA SLC7A11-C414A was from Hanbio. Transfections were performed with Lipofectamine 3000 (L3000008; Invitrogen, US). LM3 cells were infected with luciferase-expressing lentiviruses (Public Protein/Plasmid Library, CHN) and selected with puromycin (A1113803, Gibco, US).

**Cell counting kit 8 (CCK-8) assays**

Cell viability was detected using the Cell Counting Kit-8(Dojindo Laboratories, JPN). The average percentage of inhibition at each concentration and growth at a different time (0h, 24h, 48h, 72h) were calculated.

**RNA Extraction and qPCR Assays**

Total RNA was extracted from cultured cells or tissues using RNAeasy™ RNA Isolation Kit with Spin Column (R0027, Beyotime, CHN) and reverse transcribed to cDNA with a PrimeScript RT Master Mix Perfect Real-Time kit (RR037A, Takara, CHN). Subsequently, Real-Time PCR was performed with a standard SYBR Green PCR kit (RR820A, Takara, CHN). All reactions were run in triplicate, and results were normalized to β-ACTIN expression. Gene relative expression levels were derived by the 2^−ΔΔCt^ method. The primers for mice identification are 5’-AGGATGGAGTCTCGCTGTATTGC-3’ (DUXAP8 F), 5’-GGAGGTTTGTTTTCTTCTTTTTT-3’ (DUXAP8 R), 5’-TTACCAGCTTTTGTACGAGTCT-3’ (SLC7A11 F), 5’-GTGAGCTTGCAAAAGGTTAAGA-3’ (SLC7A11 R), 5’-CCTGGCACCCAGCACAAT-3’ (β-ACTIN F), 5’- GGGCCGGACTCGTCATAC -3’.

**Western Blot**

Total protein was lysed and extracted with RIPA buffer (P0013B, Beyotime, CHN). An equal amount of protein was dissolved and separated with 10% sodium dodecyl sulfate‐polyacrylamide gel electrophoresis and then transferred to polyvinylidene difluoride (PVDF) membranes (Millipore, Billerica, USA). PVDF membranes were blocked with QuickBlock™ Blocking Buffer (P0252, Beyotime, CHN) for 15 min. Membranes were subsequently incubated with primary antibody at 4℃ overnights. Having been washed three times, the blots were incubated with the appropriate amount of secondary antibodies for 1 hour at RT. The immunoreactivity was detected by enhanced chemiluminescent reagents (32132, Thermosicentific, US).

**Cell Migration and Invasion Assays**

Transwell plates (Corning Costar Corp, US) evaluated the migratory and invasive abilities. Briefly, cell suspension of each group was plated in the upper chambers of Transwell containing 200 µl serum-free DMEM medium, and 600 µl 10% FBS DMEM medium was added to the lower chamber as a chemo-attractant. After 48h of incubation, the through cells were fixed with 4% paraformaldehyde (PFA) and stained with crystal violet. For the cell invasion assay, transwell membranes were pre-coated with Matrigel (BD Biosciences, US). Each cell group was seeding to chambers after solidifying the gel. Then be performed as indicated above. The stained cells on the lower surface of the membrane were counted and imaged using a microscope (Leica, US) in 5 randomly selected fields.

**Cell colony formation assays**

After transfection, 2 ml of cell mixture containing 1 ×10^3^ cells in culture medium were seeded into six‐well plates, and the medium was replaced every other 4 days. After 2-3 weeks of incubation, cells were fixed with 4% paraformaldehyde and stained with crystal violet. Following staining, the clone cells were imaged and counted.

**Flow Cytometry**

Cell reactive oxygen species (ROS) was assessed using a ROS assay kit (R252, Dojindo, JPN). Cells were treated, harvested, and then stained with DCFH-DA following the manufacturer's instructions. Cell samples were analyzed on the flow cytometer (Beckman Instruments, US).

**Malondialdehyde (MDA) assay**

The Malondialdehyde Assay kit (Beyotime, CHN) was used to measure the amount of MDA in cell lysates following the manufacturer's protocol. Briefly, cell lysates were mixed with a reaction mixture containing thiobarbituric acid (TBA) to generate an MDA-TBA adduct. AT THE END OF THE EXPERIMENT, the MDA-TBA adduct level was calculated about the standard curve and then normalized to the total protein expression determined by BCA assays.

**Iron assay**

The free iron concentration in living cells was assessed according to the manufacturer’s instructions using the FerroOrange (F374, Dojindo, JPN). This novel ﬂuorescent probe enables live-cell ﬂuorescent imaging of intracellular Ferrous iron.

**Glutathione assay**

The GSSG/GSH Assay Kit (G262, Dojindo, JPN) was used for these experiments. The relative GSH and GSSG concentrations in cell lysates were assessed according to the manufacturer's instructions. The absorbance at 405/415 nm was measured to calculate their standard curves' GSH and GSSG amount.

**Measurement of lipid ROS**

According to the manufacturer's instructions, the peroxide-sensitive fluorescent probe Liperfluo (L248, Dojindo, JPN) was used to measure intracellular lipid ROS.

**Transmission electron microscopy (TEM)**

Cells were fixed with a solution containing 2.5% glutaraldehyde for 5 minutes, then scraped off, collected, and stained en bloc with 1% Millipore-filtered uranyl acetate. After dehydrating and embedding, the Leica EM UC7 microtome was used to cut the ultrathin sections, last, examined with a HITACHI HT7700 transmission electron microscope at an accelerating voltage of 80kV under 1.5k,6k, and 12k magnification.

**RNA immunoprecipitation (RIP)**

The RIP assay was performed using the Imprint® RNA Immunoprecipitation Kit (Sigma-Aldrich, US), following the manufacturer's instructions. Cells lysates were incubated with anti-SLC7A11 and IgG antibodies. Input RNA and immunoprecipitated RNA were extracted and purified, then detected by qRT-PCR using specific primers for DUXAP8.

**RNA pull-down assay**

For biotinylated RNA pull-down assays, DUXAP8 and its antisense RNA were transcribed in vitro using Ribo™ RNA max-T7 Biotin Transcription Kit (C11002-1, RiboBio, CHN). Then, DUXAP8 and antisense were end-labeled with desthiobiotin using a Pierce RNA 3′ End Desthiobiotinylation Kit (20163, Thermo Scientific, US). Biotin-labeled DUXAP8 were captured with streptavidin magnetic beads and incubated with whole-cell protein lysates for immunoprecipitation using Pierce™ Magnetic RNA-Protein Pull-Down Kit (20164, Thermo Scientific, US).

**Immunofluorescence staining (IF)**

Tissue samples were embedded in OCT and cut into 5 µm sections, washed in PBS, blocked with BSA, incubated at 4°C overnight with primary antibodies against SLC7A11 (1: 100) universal antibody diluent, and incubated with appropriate secondary antibodies for 1h. Finally, the sections were observed under laser confocal microscopy (Leica, US).

**Hematoxylin & Eosin staining(H&E)**

The tissues were fixed in 4% PFA solution, then embedded in paraffin, cut into 5 µm sections, and stained by Hematoxylin and Eosin Staining Kit (C0105S, Beyotime, CHN). Subsequently, the sections were imaged under a microscope.

**Tissue microarray and In situ hybridization (ISH) analysis**

Tumour tissues and the corresponding adjacent normal tissues were fixed, embedding tissue microarray and sectioning (thickness of 4μm). The expression of DUXAP8 was detected by Enhanced Sensitive ISH Detection kitⅠ (MK1030, BOSTER, CHN) according to the manufacturer's instructions. The RNAscope probe targeting DUXAP8 was designed and synthesized by Servicebio company. The results of IHC staining were evaluated automatically by with IHC Profiler plugin of software ImageJ (National Institutes of Health, Bethesda, Maryland). Each component immunoreactivity was measured on three different representative slide areas randomly selected and further divided into four groups based on the number of positive cells: high positive, positive, low positive and negative. The following algebraic formula was used to calculate the IHC optical density score (from 1 to 4) for the IHC images. IHC optical density score (OD) = (percentage contribution of high positive ×4+ percentage contribution of positive ×3+ percentage contribution of weak positive ×2+ percentage contribution of negative ×1)/100.

**Supplementary Figures**


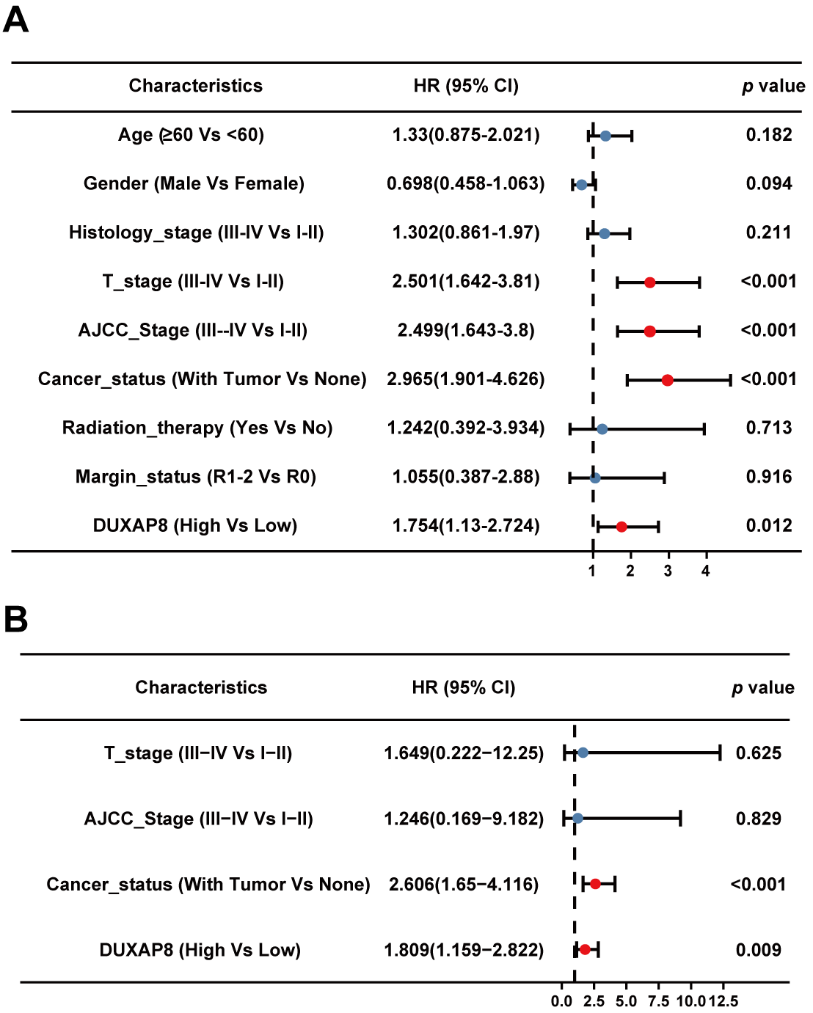
**Figure S1 Correlations of clinicopathologic characteristics with prognosis of HCC patients (n=368, TCGA database).**

**(A)** Univariate cox proportional hazards analysis. **(B)** Multivariate cox proportional hazards analysis. HR: pooled hazard ratio.


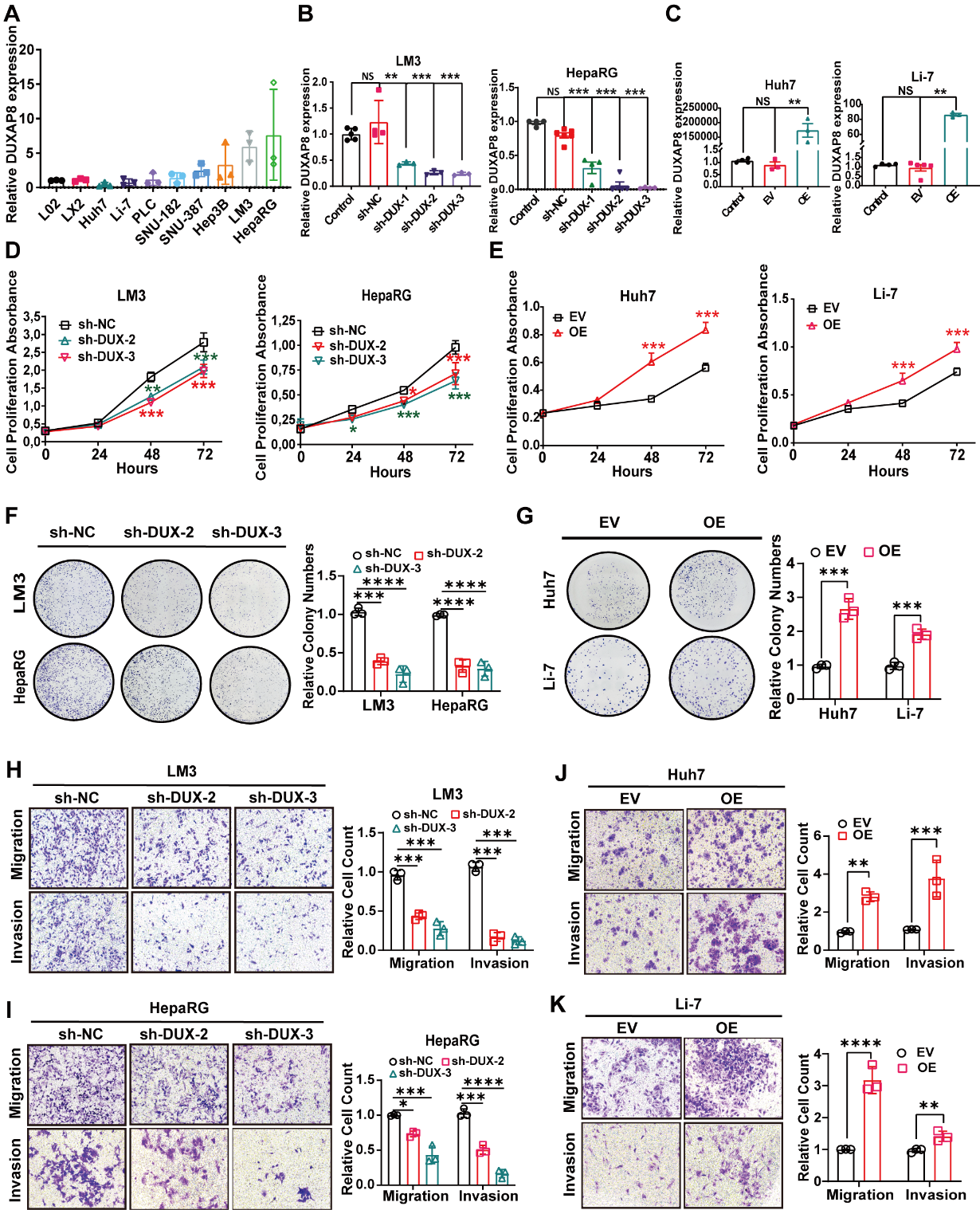
**Figure S2 DUXAP8 plays an oncogenic role in HCC cells.**

**(A)**Relative DUXAP8 expression levels detected by PCR in normal liver and HCC cell lines. **(B)** Relative DUXAP8 expression levels in LM3 and HepaRG cells transfected with shRNAs(sh-DUXAP8-1/2/3). **(C)** Relative DUXAP8 expression levels in Huh7 and Li-7 cells transfected with pcDNA DUXAP8. **(D, E)** Cell proliferation detected by CCK-8 assay after sh-DUXAP8#2/3 or pcDNA DUXAP8 transfection. **(F, G)** Colony formation of HCC cells after sh-DUXAP8#2/3 or pcDNA DUXAP8 transfection. **(H, I)** Migration and invasion of LM3 and HepaRG cells detected by transwell culture after DUXAP8 knockdown. **(J, K)** Migration and invasion of Huh7 and Li-7 cells after DUXAP8 overexpression. * *p <*0.05, ** *p <*0.01, *** *p <*0.001, **** *p <*0001. NS, not significant. Results represent three independent experiments. sh-DUX: sh-DUXAP8; EV: empty vector; OE: pcDNA DUXAP8 overexpression.

**
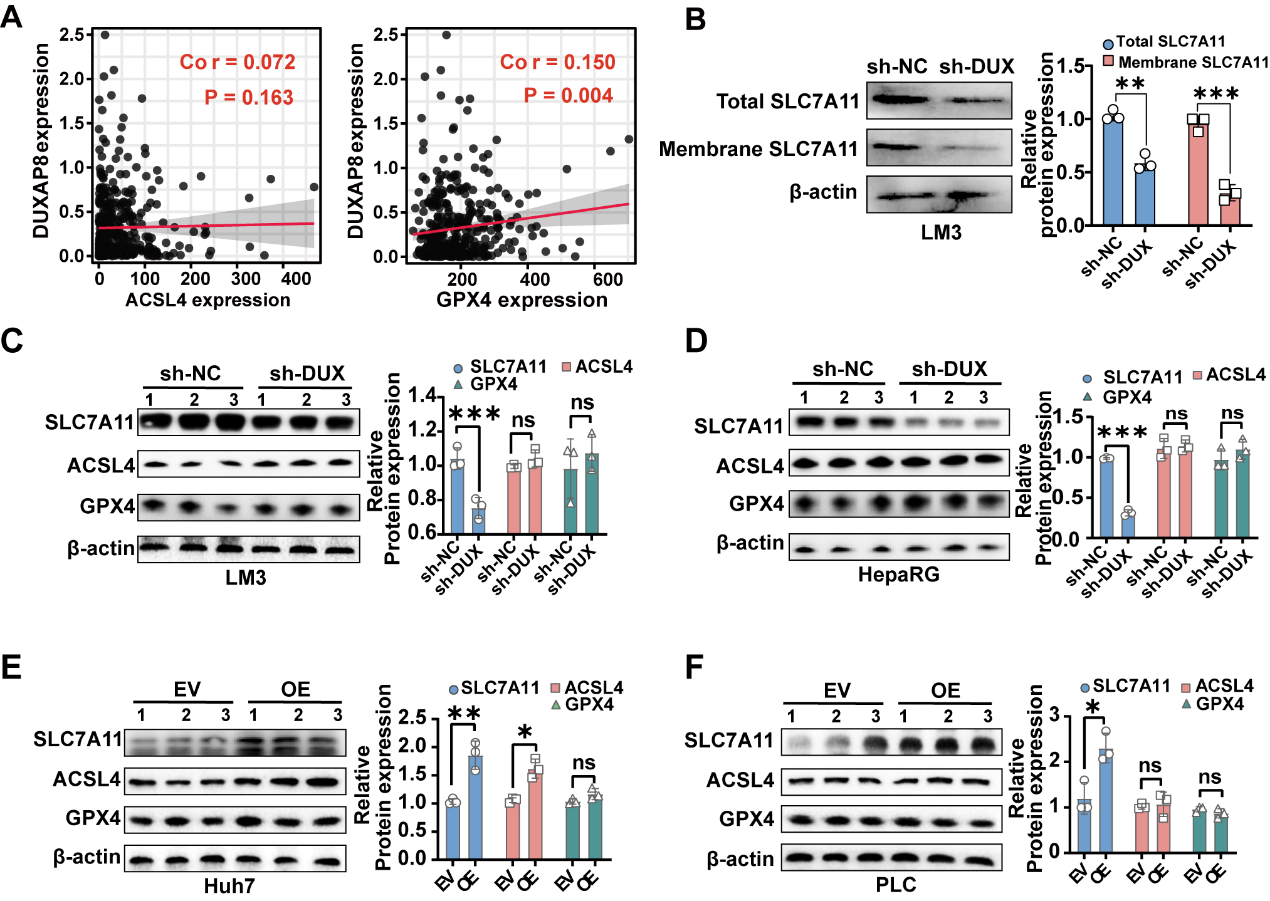
Figure S3 The link between lncRNA DUXAP8 and ferroptosis.**

**(A)** The expression of LncRNA DUXAP8 was correlated with ACSL4 and GPX4 mRNA. **(B)** The expression of LncRNA DUXAP8 was correlated with the expression of total SLC7A11 and membrane SLC7A11 protein. **(C-F)** Comparisons of SLC7A11, ACSL4, and GPX4 expression in LM3 and HepaRG cells with and without DUXAP8 knockdown and in Huh7 and PLC cells with and without DUXAP8 overexpression. * *p <*0.05, ** *p <*0.01, *** *p <*0.001. NS, not significant. Abbreviations: sh-DUX: sh-DUXAP8; EV: Empty vector; OE: pcDNA DUXAP8.

**
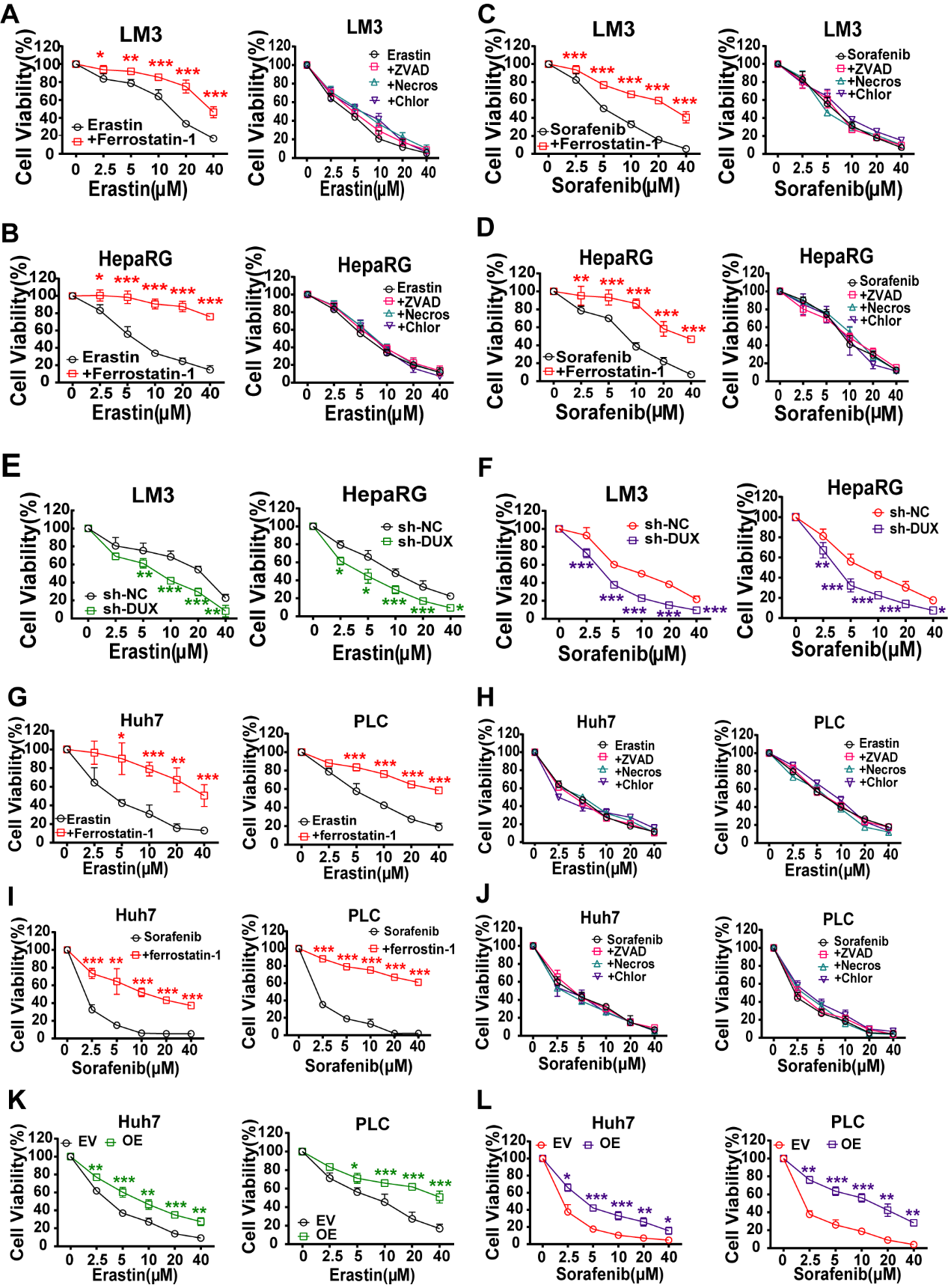
Figure S4 Erastin and sorafenib induced ferroptosis in HCC cells.**

**(A, B)** The LM3 and HepaRG cells were treated with erastin (0,2.5,5,10,20,40 µM) with or without a cell death inhibitor (ferroptosis inhibitor: ferrostatin-1,1 µM; apoptosis inhibitor: ZVAD-FMK,10 µM; necrosis inhibitor: necrosulfonamide, 0.5 µM; autophagy inhibitor: Chloroquine,10 µM) for 24 hours. Cell death was assayed using a CCK8 kit. **(C, D)** The LM3 and HepaRG cells were treated with sorafenib (0,2.5,5,10,20,40 µM) with or without a cell death inhibitor (ferrostatin-1, 1 µM; ZVAD-FMK, 10 µM; necrosulfonamide, 0.5 µM; Chloroquine,10 µM) for 24 hours. Cell death was assayed using a CCK8 kit. **(E, F)** Knockdown of DUXAP8 increased erastin and sorafenib induced cell death in LM3 and HeoaRG cells. Indicated cells were treated with erastin or sorafenib (0,2.5,5,10,20,40 µM) for 24 hours. Cell death was assayed using a CCK8 kit. **p <*0.05, ***p<*0.01, ****p<*0.001. **(G, H)** The effect of erastin and sorafenib or in combination with other cell death inhibitors on the inhibition of growth of Huh7 and PLC cells. Cells were treated with erastin (0,2.5,5,10,20,40 µM) with or without a cell death inhibitor (ferrostatin-1, 1 µM; ZVAD-FMK, 10 µM; necrosulfonamide, 0.5 µM; Chloroquine,10 µM) for 24 h, and CCK8 assayed the inhibition of growth. **(I, J)** Huh7 and PLC cells cells were treated with sorafenib (0,2.5,5,10,20,40 µM) with or without a cell death inhibitor (ferrostatin-1, 1 µM; ZVAD-FMK, 10 µM; necrosulfonamide, 0.5 µM; Chloroquine,10 µM) for 24 hours. Cell death was assayed using a CCK8 kit. **(K, L)** Increasing of DUXAP8 enhanced cell death in Huh7 and PLC cells. Indicated cells were treated with erastin (0,2.5,5,10,20,40 µM) or sorafenib (0,2.5,5,10,20,40 µM) for 24 hours. Cell death was assayed using a CCK8 kit. **p <*0.05, ** *p <*0.01, ****p <*0.001. Results represent three independent experiments. Abbreviation: sh-DUX, sh-DUXAP8; EV, Empty vector; OE, pcDNA DUXAP8; ZVAD, ZVAD-FMK; Nercros, Nercrosulfonamide; Chlor, Chloroquine.

**
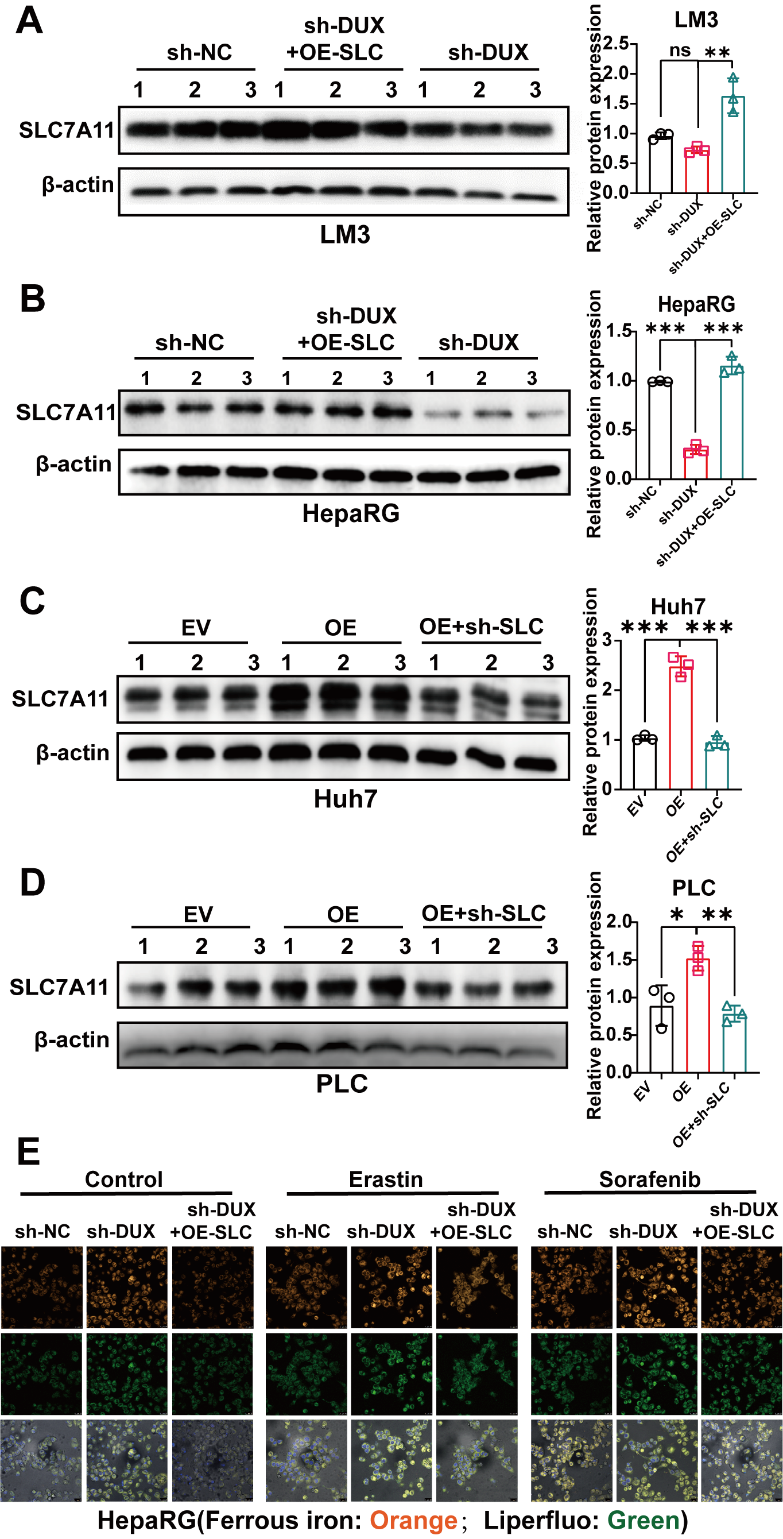
**

**Figure S5 SLC7A11 rescued DUXAP8 mediated ferroptosis.**

**(A,B)** Immunoblot analysis of SLC7A11 in LM3 and HepaRG cells following indicated transfections. β-actin was used as a loading control. **(C,D)** Western blot analysis of SLC7A11 in Huh7 and PLC cells following indicated transfection sh-SLCA11 after treated with pcDNA DUXAP8. β-actin was used as a loading control. **(E)** Lipid ROS levels (green) and Fe^2+^ (orange) content of indicated cells were measured by IF using Liperfluo and FerroOrange. HepaRG cells were transfected with indicated constructs and treated with erastin (10 µM) or sorafenib (5 µM). Magnification: ×400. **p <*0.05, ** *p <*0.01, *** *p <*0.001. NS, not significant. Results represent three independent experiments. Abbreviation: sh-DUX, sh-DUXAP8; EV, Empty vector; OE, pcDNA DUXAP8; OE-SLC, pcDNA SLC7A11; sh-SLC, sh-SLC7A11.

**
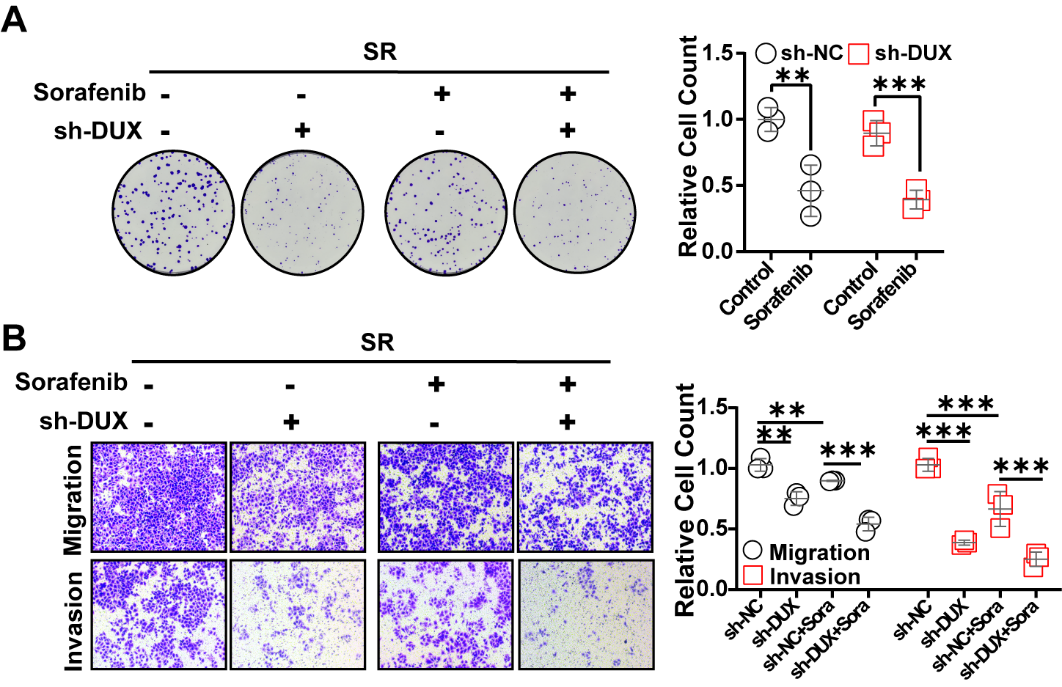
Figure S6 DUXAP8 is important for maintenance of sorafenib resistance.**

**(A)** Effect of DUXAP8 knockdown on sorafenib’s killing effect in SMMC-7721-WT and SR cell lines evaluated by colony formation assays. Cells were all treated with sorafenib (5 μM) for 24 h and cultured in complete media for another 14 days. **(B)** Brightfield images showing the effect of DUXAP8 knockdown on cell migration and invasion of SMMC-7721-SR and SMMC-7721-WT cells treated with and without sorafenib (5 μM) for 48 h. Magnification: ×100. **p <0.01, ***p <0.001. NS, not significant. Results represent three independent experiments. Abbreviation: sh-DUX, sh-DUXAP8.

**
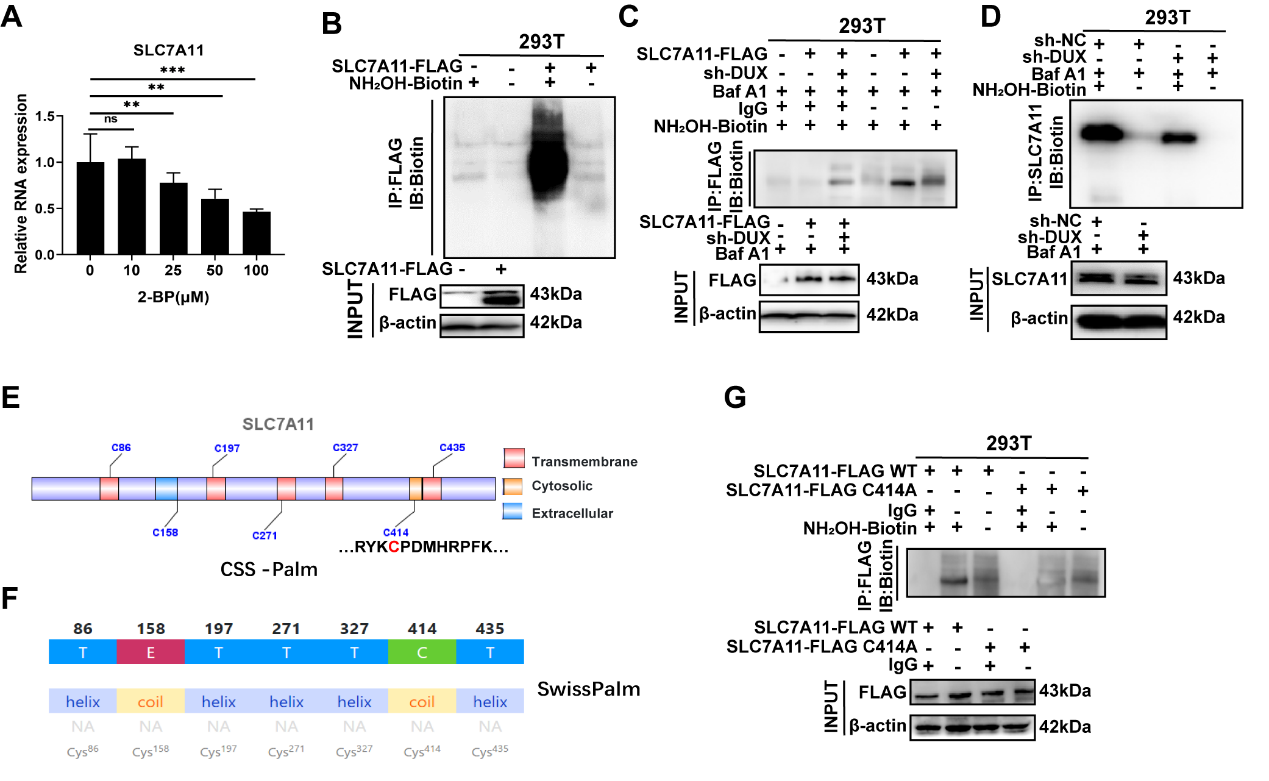
Figure S7 DUXAP8 affects SLC7A11 palmitoylation.**

**(A)** SLC7A11 mRNA expression (detected by qRT-PCR) was inhibited by 2-BP in a dose-dependent manner in LM3 cells. The cells were treated with 2-BP for 24 h. **(B)** ABE assay of 293T cells transfected with Flag-SLC7A11 plasmids. **(C)** Detection of exogenous SLC7A11 palmitoylation by ABE assays in 293T cells with and without DUXAP8 knockdown. **(D)** Detection of endogenous SLC7A11 palmitoylation by ABE assays in 293T cells with and without DUXAP8 knockdown. **(E)** Six palmitoylation sites of SLC7A11 was predicted by the CSS-palm 4.0 predictor. **(F)** Prediction of SLC7A11 palmitoylation sites by using the Swiss-Palm algorithm. **(G)** 293T cells overexpressing SLC7A11-Flag or the C414A-Flag mutant were cultured with or without NH2OH were then prepared for the ABE method and then prepared for ABE reaction. ** *p <*0.01, *** *p <*0.001. NS, not significant. Results represent three independent experiments. Abbreviation: IP, immunoprecipitation; IB, immunoblotting.

**
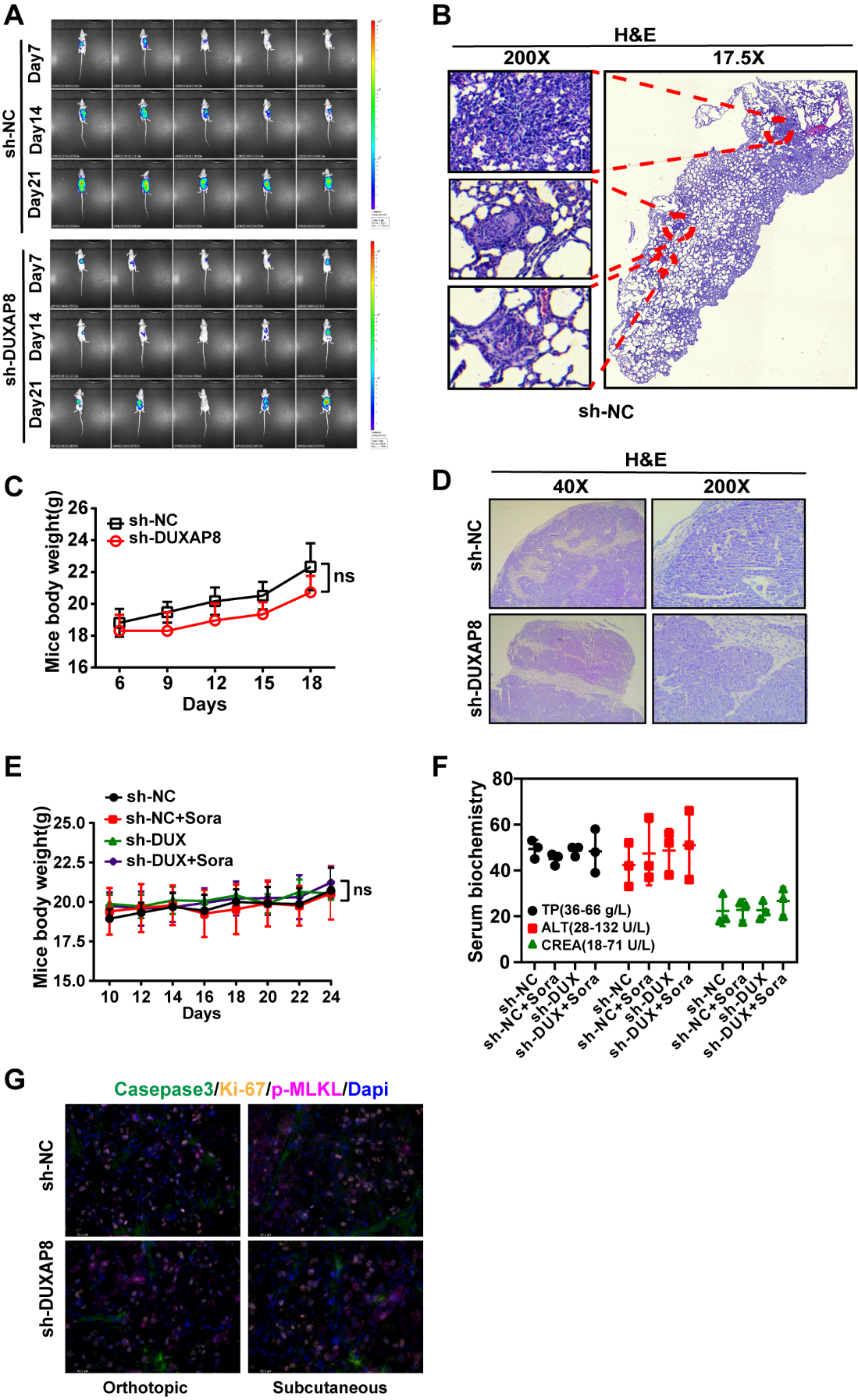
Figure S8 Knockdown DUXAP8 suppresses HCC tumor growth and metastasis in vivo.**

**(A)** Luciferase signal intensities of the mice in each group after intrahepatic injection at the indicated time points. **(B)** HCC tumor lung metastasis was found in the sh-NC group of orthotopic CDX model. Magnification: ×200 (left) and ×17.5 (right). **(C)** Body weight of the two groups of subcutaneous CDX mice. **(D)** Representative H&E staining of xenograft tumor sections. Magnification: ×40 (top panel) and ×200 (bottom panel). **(E)** Body weight of the four groups of subcutaneous SR CDX mice. **(F)** Serum Total Protein (TP), creatinine (CREA) and alanine aminotransferase (ALT) were measured in each group of subcutaneous SR CDX models. **(G)** Four-colour, multiplex immunofluorescent (mIHC) images of HCC orthotopic and subcutaneous CDX tissues sections demonstrating the proliferation, apoptosis and necrosis markers within the tumor regions. Casepase3 (green), p-MLKL (magenta), Ki-67 (yellow), and DAPI (blue). NS, not significant. Abbreviation: sh-DUX, sh-DUXAP8.

| **Characteristics** | Whole cohort (n=374) |
| --- | --- |
| **Gender** |  |
| Male | 253 (67.65%) |
| Female | 121 (32.35%) |
| **Age** |  |
| <60 | 170 (45.45%) |
| ≥60 | 204 (54.54%) |
| **Grade** |  |
| G1-2 | 233 (62.30%) |
| G3-4 | 136 (36.36%) |
| Unknow | 5 (1.34%) |
| **T stage** |  |
| TI-II | 278 (74.33%) |
| TIII-IV | 93 (24.87%) |
| Unknow | 3 (0.80%) |
| **N stage** |  |
| N0 | 254 (67.91%) |
| N1 | 4 (1.07%) |
| Unknow | 116 (31.02%) |
| **M stage** |  |
| M0 | 268 (71.66%) |
| M1 | 4 (1.07%) |
| Unknow | 102 (27.27%) |
| **AJCC stage** |  |
| I-II | 260 (69.52%) |
| III-IV | 90 (24.06%) |
| Unknow | 24 (6.42%) |
| **Cancer status** |  |
| Tumor free | 202 (54.01%) |
| With tumor | 153 (40.91%) |
| Unknow | 19 (5.08%) |
| **Margin status** |  |
| R0 | 327 (87.43%) |
| R1-2 | 18 (4.81%) |
| Unknow | 29 (7.76%) |
| **Vascular invasion** |  |
| Macro | 16 (4.28%) |
| Micro | 94 (25.13%) |
| None | 208 (55.61%) |
| Unknow | 56 (14.97%) |
| **Tumor recurrence status** |  |
| Extrahepatic Recurrence  Intrahepatic Recurrence  Locoregional Recurrence  New Primary Tumor | 23 (6.15%)  66 (17.65%)  48 (12.83%)  9 (2.41%) |
| None | 4 (1.07%) |
| Unknow | 224 (59.89%) |
| **Survival status** |  |
| Dead | 130 (34.76%) |
| Alive | 238 (63.64%) |
| Unknow | 6 (1.60) |

**Table S1** Clinicopathologic characteristics of patients with hepatocellular carcinoma from TCGA

| **Genes** | **LYSOSOME** | **Correlation coefficient** | **P value** | **Regulation** |
| --- | --- | --- | --- | --- |
| DUXAP8 | BCL10 | 0.445134 | 1.33E-19 | postive |
| DUXAP8 | GALC | 0.286925 | 1.61E-08 | postive |
| DUXAP8 | TOM1L1 | 0.137574 | 0.007714 | postive |
| DUXAP8 | RAMP3 | -0.2064 | 5.78E-05 | negative |
| DUXAP8 | DNASE2 | 0.310285 | 8.65E-10 | postive |
| DUXAP8 | HPS4 | 0.248071 | 1.19E-06 | postive |
| DUXAP8 | RAB9A | 0.144334 | 0.005164 | postive |
| DUXAP8 | TPP1 | 0.216846 | 2.34E-05 | postive |
| DUXAP8 | VAMP4 | 0.22692 | 9.35E-06 | postive |
| DUXAP8 | RAB7A | 0.357022 | 1.10E-12 | postive |
| DUXAP8 | TRIM23 | 0.179269 | 0.000495 | postive |
| DUXAP8 | CLN5 | 0.146026 | 0.004659 | postive |
| DUXAP8 | NAGLU | -0.12334 | 0.017011 | negative |
| DUXAP8 | NEU4 | -0.16227 | 0.001641 | negative |
| DUXAP8 | CTSC | 0.255318 | 5.61E-07 | postive |
| DUXAP8 | ARSB | 0.151182 | 0.003381 | postive |
| DUXAP8 | SRGN | 0.152448 | 0.00312 | postive |
| DUXAP8 | USP4 | 0.314551 | 4.93E-10 | postive |
| DUXAP8 | USP5 | 0.238342 | 3.14E-06 | postive |
| DUXAP8 | NPC2 | 0.244252 | 1.75E-06 | postive |
| DUXAP8 | GLA | 0.174946 | 0.000678 | postive |
| DUXAP8 | PPT1 | 0.39629 | 1.63E-15 | postive |
| DUXAP8 | AP3M1 | 0.219865 | 1.78E-05 | postive |
| DUXAP8 | CD63 | 0.188121 | 0.000254 | postive |
| DUXAP8 | LAMP2 | 0.111804 | 0.030638 | postive |
| DUXAP8 | SLC17A5 | 0.112801 | 0.029173 | postive |
| DUXAP8 | ANKRD27 | 0.295133 | 5.93E-09 | postive |
| DUXAP8 | VPS45 | 0.315663 | 4.25E-10 | postive |
| DUXAP8 | TIAL1 | 0.206394 | 5.78E-05 | postive |
| DUXAP8 | CORO1A | 0.127744 | 0.013424 | postive |
| DUXAP8 | HPS1 | 0.105843 | 0.040776 | postive |
| DUXAP8 | HYAL2 | 0.127584 | 0.013542 | postive |

**Table S2** The correlation of LncRNA DUXAP8 with lysosome genes
